# CD24 tracks divergent pluripotent states in mouse and human cells

**DOI:** 10.1038/ncomms8329

**Published:** 2015-06-16

**Authors:** Nika Shakiba, Carl A. White, Yonatan Y. Lipsitz, Ayako Yachie-Kinoshita, Peter D Tonge, Samer M. I. Hussein, Mira C. Puri, Judith Elbaz, James Morrissey-Scoot, Mira Li, Javier Munoz, Marco Benevento, Ian M. Rogers, Jacob H. Hanna, Albert J. R. Heck, Bernd Wollscheid, Andras Nagy, Peter W Zandstra

**Affiliations:** 1 Institute of Biomaterials and Biomedical Engineering (IBBME), University of Toronto, Toronto, Ontario, Canada M5S 3E1; 2 The Donnelly Centre for Cellular and Biomolecular Research (CCBR), University of Toronto, Toronto, Ontario, Canada M5S 3E1; 3 Lunenfeld-Tanenbaum Research Institute, Mount Sinai Hospital, Toronto, Ontario, Canada M5G 1X5; 4 Department of Medical Biophysics, University of Toronto, Toronto, Ontario, Canada M5T 3H7; 5 Biomolecular Mass Spectrometry and Proteomics, Bijvoet Center for Biomolecular Research and Utrecht University for Pharmaceutical Sciences, Utrecht University, Padualaan 8, 3584 CH Utrecht, The Netherlands; 6 Netherlands Proteomics Centre, Padualaan 8, 3584 CH Utrecht, The Netherlands; 7 Department of Physiology, University of Toronto, Toronto, Ontario, Canada M5S 1A8; 8 Department of Obstetrics and Gynecology, University of Toronto, Toronto, Ontario, Canada M5G 1E2; 9 The Department of Molecular Genetics, Weizmann Institute of Science, Rehovot, Israel 76100; 10 Department of Biology, Institute of Molecular Systems Biology, Swiss Federal Institute of Technology (ETH) Zürich, Wolfgang-Pauli Strasse 16, 8093 Zürich, Switzerland; 11 NCCR Neuro Center for Proteomics, University and Swiss Federal Institute of Technology (ETH) Zürich, Wolfgang-Pauli Strasse 16, 8093 Zurich, Switzerland; 12 Department of Health Sciences and Technology, Swiss Federal Institute of Technology (ETH) Zürich, Universitätstrasse 2, 8092 Zurich, Switzerland; 13 Institute of Medical Science, University of Toronto, Toronto, Ontario, Canada M5T 3H7

## Abstract

Reprogramming is a dynamic process that can result in multiple pluripotent cell types emerging from divergent paths. Cell surface protein expression is a particularly desirable tool to categorize reprogramming and pluripotency as it enables robust quantification and enrichment of live cells. Here we use cell surface proteomics to interrogate mouse cell reprogramming dynamics and discover CD24 as a marker that tracks the emergence of reprogramming-responsive cells, while enabling the analysis and enrichment of transgene-dependent (F-class) and -independent (traditional) induced pluripotent stem cells (iPSCs) at later stages. Furthermore, CD24 can be used to delineate epiblast stem cells (EpiSCs) from embryonic stem cells (ESCs) in mouse pluripotent culture. Importantly, regulated CD24 expression is conserved in human pluripotent stem cells (PSCs), tracking the conversion of human ESCs to more naive-like PSC states. Thus, CD24 is a conserved marker for tracking divergent states in both reprogramming and standard pluripotent culture.

## Abstract (web summary)

Characterizing the cellular stages that lead to induced reprogramming is of much interest and cell surface markers could offer unique advantages for this. Here the authors use surface proteomics and discover CD24 as a marker that tracks reprogramming-responsive cells and enables the analysis and enrichment of transgene-dependent and -independent induced pluriopotent stem cells.

## Graphical abstract

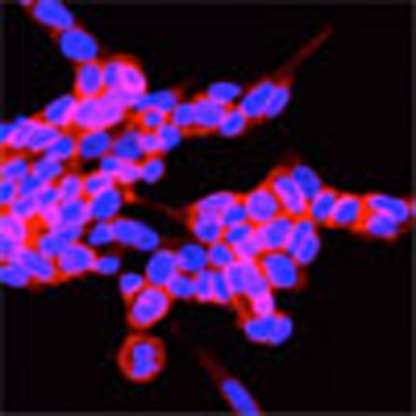

Exogenous overexpression of four key transcription factors—Oct4, Klf4, c-Myc and Sox2 (OKMS)—allows somatic cells to be induced to a pluripotent state^1^,^2^. The induced pluripotent stem cells (iPSCs) that emerge as a result of reprogramming are able to contribute to all three germ layers and give rise to an adult organism^1^. Analysis of the reprogramming time course has revealed checkpoints through which cells traverse at the genomic^3^,^4^, proteomic^5^,^6^ and epigenetic^4^ levels to achieve a final iPSC state. In the mouse system, SSEA1 is a widely used marker to track the initiation of reprogramming, Nanog and Oct4 for maturation, and Pecam1 to signify stabilization in an iPSC state^3^,^4^. Important hallmarks of successful reprogramming include the ability to silence transgenes and the ability to give rise to all germ layers on differentiation^1^,^7^,^8^.

While surrogate markers have been used to track the emergence of embryonic stem cell (ESC)-like iPSCs during reprogramming, not all cells traverse common checkpoints to attain a final transgene-independent pluripotent cell state^4^,^8^. In fact, it has been shown that OKMS factor expression levels play a role in directing cell fate changes during reprogramming. Recently, Tonge *et al.*^9^ showed that high levels of OKMS expression direct cells along a divergent reprogramming route to produce ‘F-class' iPSCs, a novel class of iPSC that is transgene-dependent while exhibiting pluripotent properties. This novel state provides insights into the potential spectrum of states emerging as a result of OKMS overexpression while also possessing advantageous properties over traditional, ESC-like cells. F-class iPSCs not only possess a growth advantage over ESC-like iPSCs, but their self-renewal is leukemia inhibitory factor (LIF)-independent, allowing for cost-efficient cell production^9^. Furthermore, F-class cells can be transitioned to an ESC-like iPSC state with the addition of histone deacetylase inhibitors^9^.

With growing knowledge on the role of OKMS expression levels in directing cell fate changes there is a need for easy and reliable benchmarking tools to track cell state during reprogramming. Cell surface proteins are particularly desirable as a tool because of their ability to be quantified with relative ease and without the need to sacrifice cell viability.

As part of the international Project Grandiose initiative^10^, we set out to identify surface glycoproteins emerging during high OKMS factor expression that could capture divergent cell state changes between transgene-dependent (F-class) and ESC-like iPSCs^9^. Our analysis and subsequent validation reveal CD24 as a novel candidate marker that is able to demarcate transgene-dependent (F-class) and transgene-independent (ESC-like) mouse iPSCs while identifying ‘primed' and ‘naive' pluripotent states in transgene-independent populations. Importantly, CD24 exhibits conservation in the human system, tracking the conversion of human ESCs to more naive-like alternative pluripotent states^11^, thus allowing for enrichment of these cells.

## Results

### Cell surface proteome dynamics during reprogramming

In order to investigate changes in cell surface glycoproteins resulting in high OKMS factor expression, we employed a secondary (2°), doxycycline (DOX)-inducible mouse embryonic fibroblast (MEF) 1B reprogramming system^7^,^10^. As previously described^10^, cell samples were collected at multiday intervals over the complete reprogramming time course of 30 days (cultured at 1,500 ng ml^−1^ of DOX and termed the ‘DOX-high (H)' time course), and over one derivate time course (cultured at 5 ng ml^−1^, termed ‘DOX-low (L)'), which was split from the DOX-high time course on day 8 (ref. 10). Samples were then prepared for protein analysis using mass spectrometry through enrichment for cell surface glycopeptides^12^, and labelling of ‘full' protein content for quantification^13^, reported by Benevento *et al.*^14^. A summary of the mass spectrometry pipeline is shown in Supplementary Fig. 1a.

We identified a total of 896N-glycosylated peptides, representing 432 protein groups (or ‘proteoforms'^15^) at an estimated confidence level of 98% (Supplementary Data 1 provides a deconvolution of isoforms). To the best of our knowledge, 34 of these proteoforms have not previously been definitively detected at the cell surface and 86 have not been reported in the context of ESC/iPSC. A list of amino-acid sequences for the 896 detected glycopeptides is provided with sequons highlighted as a resource addressing sites of N-glycosylation (Supplementary Data 2). Panther gene list analysis^16^,^17^ of the genes corresponding to the full set of detected surface proteins reported their division into eight functional categories (Supplementary Fig. 1b). Receptor, catalytic, binding and transporter activities proved to be dominant. The s.d. of the percentage proportion across the intervals for a given category averaged to 1.5% for all categories (Supplementary Data 3).

Raw, normalized and averaged spectral counts across three technical replicates per point of the DOX-high time course are presented in Supplementary Data 4. Results are also available on www.stemformatics.org as track files, which enables automated upload to the UCSC genome browser^18^. In order to improve the rigour of the quantitative analysis, we used only that subset of the detected cell surface proteome which overlapped those proteins detected in the ‘full' proteome^14^, namely 185 proteoforms, except where otherwise noted. Together, these are listed for further examination as potential surface markers (Supplementary Data 5).

The sharpest degree of protein change at the cell surface occurred between days 0 and 2 (Supplementary Fig. 1c). Downregulation was observed across all of the functional categories (Supplementary Data 6). The distribution of the downregulated proteins among functional categories was congruent to the proportions of the categories for the full list of detected cell surface proteins. Complementary upregulation occurred between day 18 (H) and the 2° iPSC state (Supplementary Fig. 1d), and to a lesser extent, between day 21 (DOX-low) and the 2° iPSC state. Overall, 78% of the post day 18 upregulated proteins (including most collagens and integrins) were previously downregulated twofold or more (between days 0 and 2). This indicates the impact of transgene activation in directing changes at the cell surface. Interestingly, as compared with the global proteome monitored throughout reprogramming^14^, cell surface glycoproteins are proportionally over-represented among proteins exhibiting twofold or greater change between each time point, and in proteins associated with cell signalling and cell surface receptor expression (Supplementary Data 6). Together, these results underline the importance of utilizing these proteins to track cell state changes.

**Figure 1:**
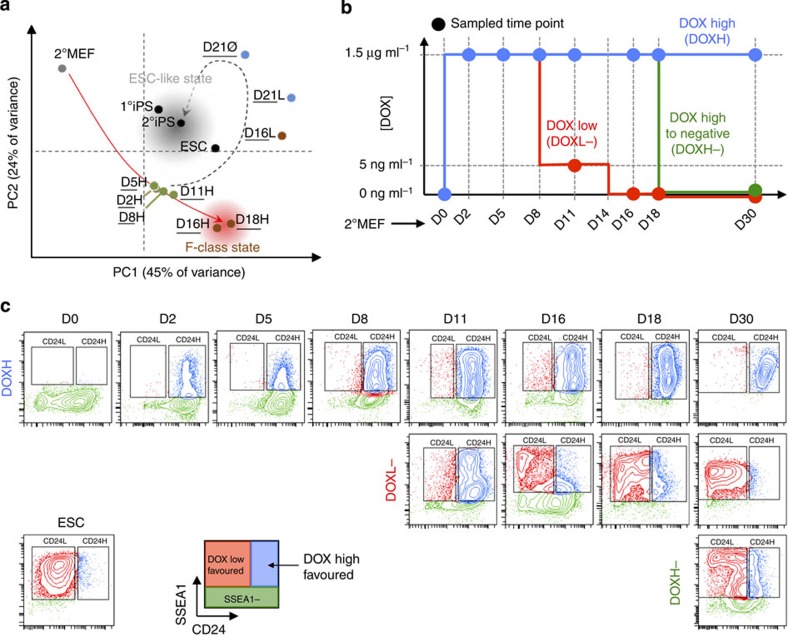
Surface proteome analysis during reprogramming identifies CD24 as a differentially expressed surface marker. (**a**) Principal component analysis of the surface proteome (including only the subset of surface proteins that were also present in the global proteome screen to improve rigour of quantitative analysis) of reprogramming secondary MEF 1B cells derived from tetraploid complementation, showing divergent routes of F-class and ESC-like iPSCs. (**b**) Summary of sampling time course and DOX treatment protocols. (**c**) Representative flow cytometry plots of CD24 versus SSEA1 expression during reprogramming of secondary MEF 1B cells derived from tetraploid complementation, revealing emerging CD24^high^/SSEA1+ (CD24H) and CD24^low^/SSEA1+ (CD24L) subpopulations. ESC control is included for comparison. Flow plots are representative from three biological replicates.

Consistent with the other ‘omic' analyses conducted as part of the Project Grandiose reprogramming analysis^10^, principal component analysis of surface proteins depicted the DOX-high time course diverging from an ESC-like state to a separate state, whereas the DOX-low time course converges on the ESC-like state (Fig. 1a). The divergence suggests the possibility of capturing an alternative reprogramming state, F-class^9^, by means of surface proteomics. We then conducted subsequent analysis to select surface proteins for additional scrutiny and validation (Supplementary Fig. 2). First, we conducted K-means clustering analysis to select genes that are differentially expressed in the F-class state, identifying two clusters of genes that are upregulated following DOX induction and maximally expressed at the F-class state (Supplementary Fig. 2). Of these genes, we selected those with the highest contribution to the principal components separating the F-class and ESC states as well as F-class and MEF states. This produced a list of genes, of which many were metabolic or neural markers. A handful of proteins were chosen based on this analysis as well as antibody availability (Supplementary Fig. 3) for validation along the reprogramming time course (Fig. 1b), which revealed CD24 as the best candidate based on its ability to differentiate between emerging F-class and ESC-like iPSCs (Fig. 1c). We next proceeded with a detailed characterization of CD24 expression during reprogramming.

### CD24 as a marker of divergent reprogramming states

Project Grandiose ‘omic' data show transcriptional upregulation of CD24 expression following DOX addition and a reduction in the ESC-like iPSC state (Supplementary Fig. 4a). Furthermore, the CD24 locus exhibits high levels of H3K4me3 and H3K36me3 activation marks during the reprogramming time course, with elevated H3K27me3 repressive mark at the final ESC-like iPSC stage^10^ (Supplementary Fig. 4a). This trend was conserved in previously published reprogramming systems^3^,^4^,^8^,^19^,^20^, where the levels of CD24 increase following reprogramming induction and downregulate at the iPSC state where ectopic transgene expression is removed (Supplementary Fig. 5). Furthermore, CD24 levels are further reduced in the Thy1+ fraction of cells as compared with the reprogramming Thy1-/SSEA1+ fractions in the Polo *et al.* data set, supporting the view that expression of CD24 can help distinguish reprogramming cells from somatic and pluripotent cell states (Supplementary Fig. 5d).

Recently, CD44 and Icam1 were used to track the progression of reprogramming MEFs as they move through CD44+/Icam1− and CD44−/Icam1−/Nanog+ states to reach a final CD44−/Icam1+ iPSC state^6^. CD44/Icam1 dynamics in the Project Grandiose data exhibited a similar trajectory, with CD44 transcriptome levels reaching maximal levels following DOX addition and decreasing as the ESC-like iPSC state is reached, consistent with the acquisition of the H3K27me3 repressive mark at the ESC-like iPSC state^10^ (Supplementary Fig. 6a). Icam1 levels decrease following DOX removal and increase as cells progress through reprogramming, reaching a maximal level at the ESC-like iPSC state where H3K27me3 repression marks are lost and H3K36me3 activation marks are gained^10^ (Supplementary Fig. 6a). In order to assess the utility of combining CD44/Icam1 and CD24 as markers to delineate divergent reprogramming populations, we next evaluated the expression of these markers on live reprogramming cells using flow cytometry.

Analysis of CD24 expression was conducted using 2°MEFs treated in the DOX-high (DOXH), DOX-low-to-negative (DOXL−) and DOX-high-to-negative (DOXH−) time course, as previously described (Fig. 1b)^10^. Importantly, CD24 expression levels showed concordance across flow cytometry and mass spectrometry platforms (Supplementary Fig. 4b). Flow cytometry for CD24/SSEA1 expression along the three DOX time courses revealed the emergence of a CD24^high^/SSEA1+ population in the DOXH condition, hereafter referred to as CD24H cells, while a CD24^low^/SSEA1+ population stabilized in the DOXL− and DOXH− conditions, hereafter referred to as CD24L cells (Fig. 1c, Supplementary Fig. 7a). The gating strategy henceforth used to define and quantify CD24H/L cells is shown in Supplementary Fig. 7b. Importantly, when the CD24/SSEA1 staining strategy was applied to a different reprogramming system, Col1a1 secondary reprogramming MEFs^21^, the utility of CD24 as a marker for tracking reprogramming is conserved (Supplementary Fig. 8a). As anticipated, DOX treatment upregulated CD24 such that nearly all cells (93.8±0.4%) were CD24^high^ by 2 days (Supplementary Fig. 8a). While this reprogramming system did not give rise to SSEA1+ cells as quickly as the 1B system, a small CD24H fraction emerged after 8 days of DOX treatment (Supplementary Fig. 8a); however, this CD24H subpopulation was largely transient and the CD24L fraction dominated (Supplementary Fig. 8a). This is consistent with the observation of Tonge *et al.*^22^ that these cells do not stabilize in an F-class state.

F-class cells are known to homogenously emerge in DOXH conditions (using the 2°MEF 1B tetraploid system) while ESC-like iPSCs are observed solely in DOX-negative (DOX−) conditions^9^,^10^. This suggested that CD24H cells (in DOXH) may correspond to F-class cells, while CD24L cells may correspond to ESC-like iPSCs. Control ESCs exhibited a CD24L staining profile, supporting this hypothesis (Fig. 1c). F-class cells derived from primary DOX-inducible 1B cells as well as tail tip fibroblasts also showed a consistent CD24H expression profile (Supplementary Fig. 8b). Interestingly, secondary MEFs derived from a 27% chimera, exhibiting different OKMS expression levels, also traverse a CD24H state following DOX induction (Supplementary Fig. 9). This observation also supports the fact that OKMS stoichiometry plays a role in directing cell fate in reprogramming^9^,^10^. In fact, a recent report shows that lower levels of Klf4 overexpression during reprogramming stalls the cells in a partially reprogrammed, transgene-dependent state^23^, which is consistent with our observation that, while MEF 1Bs derived from tetraploid complementation give rise to F-class cells robustly, 1B MEFs from 27% chimeras exhibit higher levels of Klf4 transgene (Supplementary Fig. 9c) and are able to give rise to ESC-like iPSCs in DOXH culture (Supplementary Fig. 9a).

While CD24 shows potential for demarcating divergent populations emerging from unique DOX treatment time courses, flow cytometry staining for CD44/Icam1 shows DOXH-treated cells prematurely transition to a CD44−/Icam1+ state, which is reportedly indicative of Nanog+ iPSCs^6^ (Supplementary Fig. 6b,c). Thus, CD44/Icam1 cannot demarcate F-class and ESC-like iPSCs, demonstrating the utility of CD24 for categorizing reprogramming populations. Interestingly, CD44+/Icam1+ cells maintain the highest proportions of CD24^high^ cells, while CD44-/Icam1+ cells exhibit the lowest proportion of CD24^high^ cells in intermediate reprogramming stages (Supplementary Fig. 6d). As a positive marker for the F-class state, CD24 allows for a direct demarcation of divergent reprogramming from an ESC-like state, which is well characterized and identified by surface proteins such as Pecam1, E-cadherin^3^ and CD44/Icam1 (ref. 6).

### Characterization of CD24H and CD24L subpopulations

**Figure 2:**
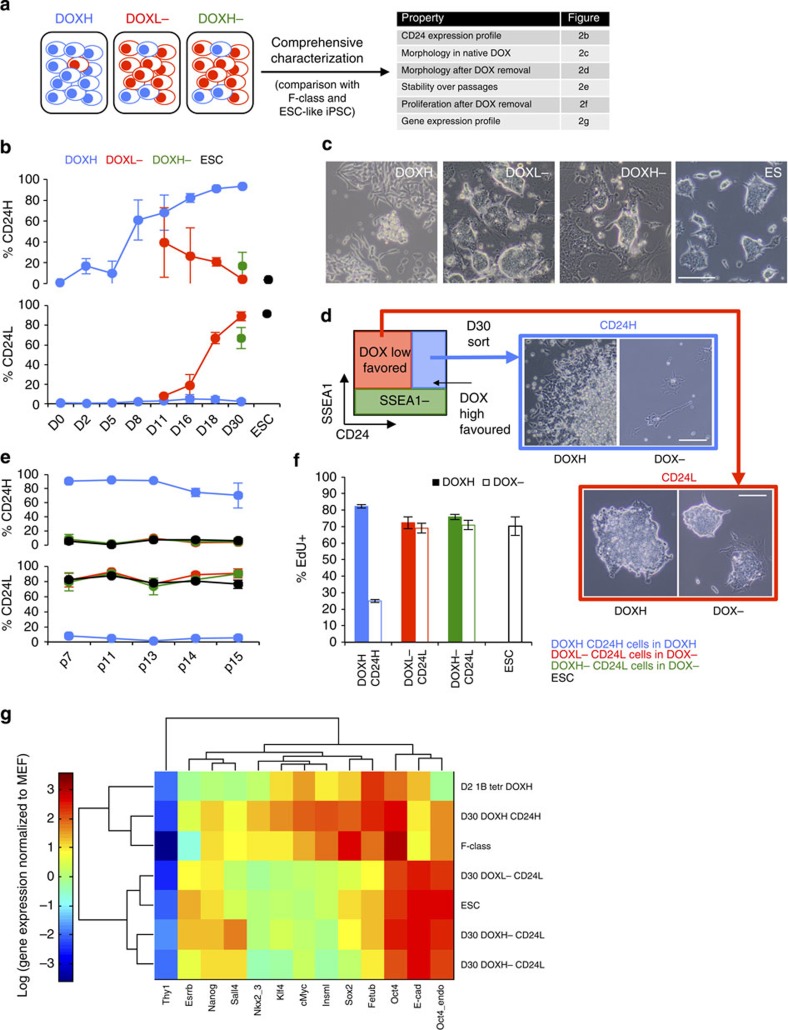
CD24H and CD24L subpopulations correspond to transgene-dependent (F-class) and ESC-like iPSCs, respectively. (**a**) Summary of approach to characterize CD24H and CD24L populations. (**b**) Percentage of CD24H and CD24L cells in DOXH, DOXL− and DOXH− culture time courses. Data bars show mean±s.d. (*n*=3 biological replicates). (**c**) Representative phase contrast images (*n*=3 biological replicates) of emerging colonies in the three DOX treatments, including ESC control. Scale bar, 125 μm. (**d**) Summary of sorting strategy used to separate CD24H and CD24L subpopulations from D30 culture. Representative phase contrast images (*n*=3 technical replicates) of sorted CD24H and CD24L cells in DOXH and DOX− conditions. Scale bar, 125 μm. (**e**) Effect of long-term passaging on levels of CD24H and CD24L cells in D30-sorted DOXH CD24H, DOXL− CD24L and DOXH− CD24L cells. ESCs are included as a control. Data bars show mean±s.d. (*n*=3 technical replicates). (**f**) EdU staining of DOXH CD24H, DOXL− CD24L and DOXH− CD24L cells in DOXH and DOX− conditions to assess DOX dependence of proliferation. Data bars show mean±s.d. (*n*=3 technical replicates). (**g**) Expression levels of various pluripotent and F-class-specific genes in CD24H and CD24L cells with unsupervised hierarchical clustering, including F-class and ESC cell controls, normalized to *Gapdh* and MEF cells.

In order to comprehensively compare CD24H and CD24L cells to F-class/ESC-like iPSCs, we chose the following criteria to characterize the newly defined cells (Fig. 2a): CD24/SSEA1 expression levels compared with ESC controls; morphology in native culture; morphology in response to DOX removal; stability following extensive passaging; dependence of proliferation on DOX; and gene expression profile.

Quantification of the percentage of CD24H and CD24L cells emerging in the DOX time courses suggested that CD24H cells arise homogenously in DOXH culture, exhibiting an F-class morphology, while CD24L cells emerge in DOXL− and DOXH− culture, exhibiting an ESC-like morphology (Fig. 2b,c, Supplementary Fig. 10a). Control ESCs expressed a CD24^low^/SSEA1+ profile, consistent with CD24L cells (Figs 1c and 2b). This trend was independent of passaging frequency (Supplementary Fig. 10b). Following sorting for the CD24H and CD24L populations from DOXH and DOXL− time courses on day 30 of reprogramming, CD24H cells maintained their F-class morphology while DOX removal resulted in the loss of these cells (Fig. 2d), consistent with reports from Tonge *et al.*^9^ On the other hand, CD24L-sorted cells maintained their ESC-like morphology and were stable in both DOXH and DOX− conditions (Fig. 2d).

Continued passaging of CD24H cells in DOXH and CD24L cells (from DOXL− and DOXH− time courses) in DOX− conditions revealed that these cells maintained their CD24 expression profiles in their native DOX conditions (Fig. 2e; Supplementary Figs 10c and 11a). Consistent with the observation of CD24H DOX dependence, removal of DOX in these cells resulted in reduced proliferation, assessed by 5-ethynyl-2′-deoxyuridine (EdU) staining (Fig. 2f). Gene expression analysis showed CD24H cells (derived from day 30 DOXH culture) clustered with F-class cells (taken from Tonge *et al.*^9^), while CD24L cells (derived from day 30 DOXL− and DOXH− culture) clustered with ESCs, confirming the ability of CD24/SSEA1 staining to demarcate F-class and ESC-like iPSC populations (Fig. 2g). As expected, ESC-like iPSC markers, Pecam1 and E-cadherin, were seen in CD24L cells while not in CD24H cells (Supplementary Fig. 11b). Furthermore, CD24L cells exhibited levels of OKMS expression that are consistent with ESCs (Fig. 2g). Consistent with Tonge *et al.*^9^, both CD24H and CD24L cells were able to contribute to the three germ layers following *in vitro* differentiation (Supplementary Fig. 12). Overall, these studies reveal that CD24 can separate the transgene-dependent F-class iPSCs from the transgene-independent ESC-like iPSCs.

### CD24 demarcates transgene-independent pluripotent states

**Figure 3:**
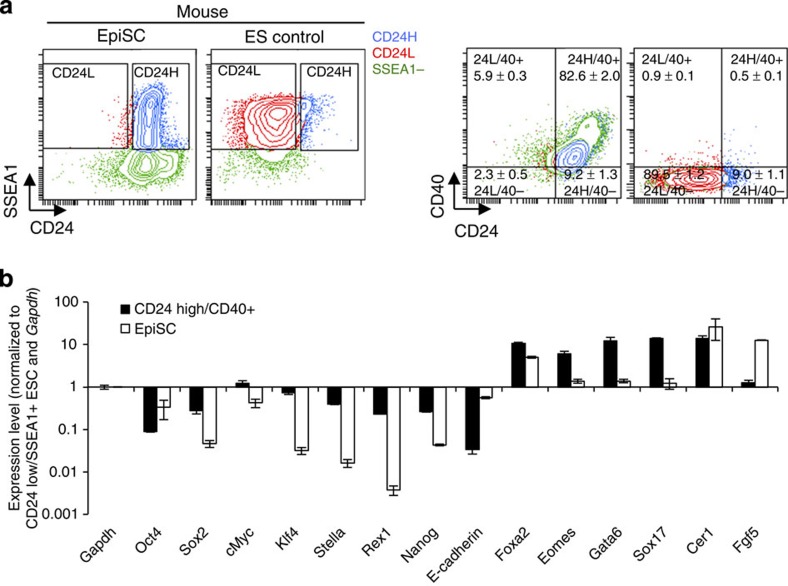
CD24 and CD40 exhibit differential expression on mouse ESCs and EpiSCs. (**a**) Flow cytometry analysis of CD24/SSEA1/CD40 expression in mouse ESCs and EpiSCs. Representative flow plots (*n*=3 technical replicates) of stained mouse ESC and control embryo-derived EpiSCs. (**b**) Gene expression of CD24^high^/CD40+ cells derived from R1 ESC culture compared with control embryo-derived EpiSCs. The gene panel is composed of pluripotency genes as well as some early differentiation markers. Values are normalized to sorted CD24^low^/SSEA1+ (ESC-like) cells sorted from R1 ESC culture and *Gapdh*. Data bars show mean±s.d. (*n*=3 technical replicates).

We have shown that CD24 can be used to demarcate transgene-dependent F-class cells from ESC-like iPSCs. Given the role of CD24 in separating these iPSC states, we wondered whether CD24 would show conservation and utility in nonreprogrammed pluripotent populations. In order to investigate this possibility, we co-stained both mouse ESC and epiblast stem cell (EpiSC) populations for CD40 (a known EpiSC surface marker^24^) and CD24. Staining for CD24/CD40/SSEA1 levels in control mouse ESCs and EpiSCs confirmed the ability of CD24^high^/CD40+ staining to identify the EpiSC state, while ESCs were CD24^low^/CD40− (Fig. 3a). In order to better characterize the rare CD24^high^ cells emerging in standard ESC populations, we cultured ESCs in serum and LIF conditions and sorted for the rare CD24^high^/CD40+ (EpiSC-like) as well as the prevalent CD24^low^/SSEA1+ (standard ESC) fraction of cells and conducted a survey of pluripotency and early differentiation gene expression^25^ (Fig. 3b). As expected, EpiSC-like CD24^high^/CD40+ cells exhibited lowered levels of naive pluripotency genes such as *Stella*, *Rex1* and *Nanog* while also exhibiting higher levels of *Foxa2*, *Eomes*, *Gata6*, *Sox17*, *Cer1* and *Fgf5* (Fig. 3b). The observation of this rare EpiSC-like fraction of cells in serum-based ESC culture has been previously observed^26^,^27^.

Importantly, CD40 staining was negative in F-class and ESC-like iPSCs derived from the DOXH or DOXH− reprogramming time courses, while some levels of CD40 expression was evident in the cells derived from the DOXL− time course (Supplementary Fig. 13).

Finally, we tested whether culture-derived EpiSCs, generated from transgene-independent iPSCs selected from our 1B reprogramming cultures as well as an ESC control, would acquire the predicted CD24^high^/CD40+ state. Importantly, all iPSC replicates as well as the ESC control were able to give rise to CD24^high^/CD40+ cells with an EpiSC-like morphology and gene expression profile, while 2i treatment abolished any rare CD24^high^/CD40+ cells that remain in serum-based culture over continued passages (Supplementary Fig. 14). Together, these data support the notion that CD24^high^ cells in transgene-free mouse pluripotent culture represent a rare fraction of EpiSC-like cells.

### CD24 expression in human PSCs

Given the hypothesized similarities between mouse EpiSCs and human ESCs, we wondered whether CD24 expression is conserved on human (h) pluripotent stem cells (PSCs)^24^. Analysis demonstrated that CD24 expression is conserved in hESCs. Thus, CD24 serves as a tool to capture the transition from low CD24 expression in starting cells to high CD24 expression following OKMS induction and movement to a human iPSC state^28^,^29^, exhibiting levels of CD24 that are comparable with hESCs and are higher than the starting somatic cells (Supplementary Fig. 15a,b). This is consistent with one hypothesis that considers hESCs as ‘primed', analogous to the mouse EpiSC state, and in contrast to the ‘naive' cell state of mouse ESCs^30^. Data mining for expression of CD24 in the developing human embryo with single-cell RNA-seq resolution^31^ revealed increasing CD24 levels as the zygote reached a post-implantation (primed) state with very low expression in the earliest (naive) embryonic states (Supplementary Fig. 15c).

**Figure 4:**
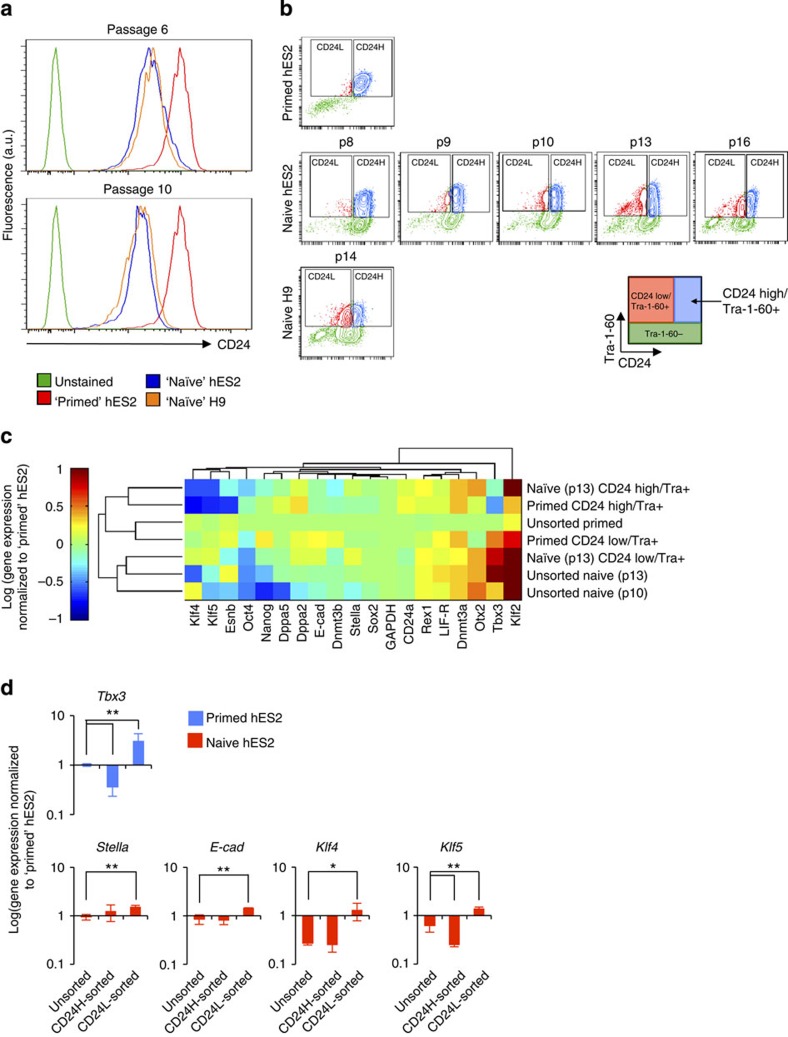
CD24 delineates 'primed' and 'naive' pluripotent states in human cells. (**a**) Flow cytometry analysis of CD24 expression in ‘primed' and ‘naive' human ESC lines following passages 6 and 10 in naive conditions. Flow plots are representative from three technical replicates. (**b**) CD24/Tra-1-60 staining of primed hES2 and naive-induced hES2 and H9 hESCs at indicated number of passages in naive conditions. Flow plots are representative from three technical replicates. (**c**) Gene expression analysis of CD24^high^/Tra-1-60+ and CD24^low^/Tra-1-60+ cells sorted from primed and naive culture at indicated passage number with unsupervised hierarchical clustering. Expression data are normalized to *GAPDH* and primed hES2 cells. (**d**) Gene expression analysis comparing unsorted, CD24H-sorted and CD24L-sorted hES2 cells. Expression data are normalized to *GAPDH* and primed hES2 cells. Statistical significance is assessed by a Student's *t*-test (heteroscedastic, two-sided). **P<0.05, *P<0.1. Data bars show mean±s.d. (*n*=3 technical replicates).

In order to test this *in vitro* we cultured hESC lines in two conditions: standard hESC culture and culture supplemented with conditions reported to induce hESCs to an alternative naive-like state^11^. Flow cytometry analysis of these cells confirmed that CD24 expression is lowered in naive-like hESC states, achieving levels equivalent with mouse ESCs by passage 10 of culture in so-called naive conditions^11^ (Fig. 4a). Further development of our naive induction strategy led us to note a dependency of CD24 downregulation on levels of LDN in our naive induction protocol (Supplementary Fig. 16a). To ensure that CD24 was indeed a reliable marker for the primed-to-naive-like state transition, we removed LDN from subsequent experiments. hESCs were co-stained for CD24/Tra-1-60 (analogous to mouse CD24/SSEA1 staining) following changes in primed-to-naive associated culture conditions (Fig. 4b). As before, an observable (although reduced in size) CD24^low^/Tra-1-60+ fraction emerged. Analysis of Oct4/Sox2 expression in naive-like cells revealed maintenance of pluripotency (Supplementary Fig. 16b). Our observation was further supported by CD24 expression data extracted from a recent study by Theunissen *et al.*^32^, reporting generation of naive hESCs without LDN (Supplementary Fig. 15d). Characterization of our naive hESCs revealed that they exhibited gene expression levels consistent with previous reports^11^,^32^,^33^. Furthermore, gene expression analysis revealed that sorted CD24^high^/Tra-1-60+ (hereafter called CD24H) cells and CD24^low^/Tra-1-60+ (hereafter called CD24L) cells clustered distinctly (Fig. 4c). Interestingly, CD24H cells derived from primed and naive-treated cultures clustered together, exhibiting especially low levels of *Tbx3* expression (Fig. 4c,d) and expectedly higher levels of *CD24a* expression (Supplementary Fig. 16c). In addition, these CD24H cells clustered with the other primed cells. On the other hand, CD24L cells from naive culture clustered with unsorted naive-treated cells. Most notably, while unsorted naive hESCs derived here showed enrichment for naive markers *Klf2*, *Tbx3*, *Otx2, Dnmt3a*, *LIF-R* and *Rex1*, CD24L-sorted naive cells showed further enrichment for naive markers *Stella*, *E-cadherin*, *Klf5* and *Klf4* (Fig. 4d). This analysis may suggest heterogeneity in these naive hESC cultures, and support CD24 as a powerful tool to resolve this heterogeneity and enrich for hPSCs in different pluripotent states.

## Discussion

**Figure 5:**
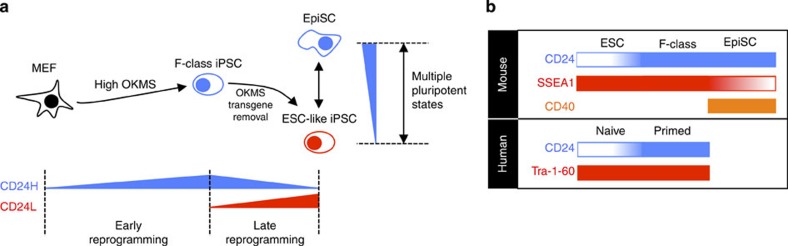
Summary of CD24 as a marker for three pluripotent states. (**a**) Overview of CD24 as a tool for tracking transgene induction in early reprogramming, demarcating divergent reprogramming to F-class and ESC-like iPSC states in late reprogramming, and identifying multiple pluripotent states in transgene-independent pluripotent culture. (**b**) Summary of approach for identifying mouse F-class iPSCs, ESC-like iPSCs and EpiSCs via CD24/SSEA1/CD40 staining as well as human primed and naive hESCs via CD24/Tra-1-60 staining.

Here we have identified CD24 as a novel cell surface marker expressed dynamically during reprogramming. CD24 serves both as a marker to demarcate transgene-dependent (F-class) and -independent (ESC-like) mouse iPSCs and for delineating naive-like and primed pluripotent states in both mouse and human (Fig. 5).

CD24, also known as heat-stable antigen, is a glycosylphosphatidylinositol-linked cell surface protein, with a core of 27 amino acids^34^. It is abundantly expressed in haematopoietic and neural cells^34^ and is a known marker for B cells^35^, pancreatic stem cells^36^ and keratinocytes, with particular expression in areas of the hair follicle containing the colony-forming cells^37^,^38^. In development, CD24 expression can be found in the primitive ectoderm, mesoderm and ventral endoderm^39^. In general, CD24 is expressed at higher levels in progenitor cells and metabolically active cells than in terminally differentiated cells^38^. CD24 has been shown to support cell adhesion of myeloid cells to P-selectin, whereby P-selectin acts as a ligand, triggering downstream src-family tyrosine kinases^34^. In the cancer literature, CD24 has been found to correlate with aggressive tumour behaviour^40^,^41^ and serves as a marker for diagnosis and prognosis^38^. For example, CD24+ hepatocellular carcinoma cells showed an increased propensity for self-renewal, differentiation and metastasis as well as enriched levels of Sox2 and Oct4 expression^40^. Importantly, knockdown of CD24 suppressed these characteristics, supporting the functional role of CD24 in tumorigenesis. Nanog has also been identified as an important downstream effector of CD24-mediated tumorigenicity and self-renewal, where CD24 phosphorylates STAT3 through src^40^. CD24-mediated c-src kinase activity has also been reported to promote integrin-mediated adhesion, epithelial-to-mesenchymal transition and invasion in breast cancer^42^,^43^. Furthermore, a recent report has shown that CD24 affects cell cycle dynamics, whereby CD24 plays a role in functionally inactivating p53 in human prostate cancer cells^44^. These reported signalling cascades may provide insight into the CD24 status in pluripotent culture as well, as there has been clear links between CD24 and core pluripotency and cell cycle regulators.

The cell surface proteome provides a means of accessing the cell state without sacrificing viability^6^. We used mass spectrometry to measure changes in surface glycoprotein^12^ expression throughout the reprogramming time course and found that cell surface proteins are markedly over-represented in proteins undergoing two- or higher fold changes in expression. This suggested that the surface proteome serves as a rich data set to identify markers that track cell state changes during reprogramming.

CD24 is upregulated quickly after reprogramming induction, reaching maximal levels within a few days of transgene overexpression in both the 1B and Col1a1 systems, with similar trends observed in other reported reprogramming systems. As a result, CD24 acts as a good marker to track the initiation of reprogramming following OKMS overexpression, during which the cells acquire a CD24^high^/SSEA1− state. In the 1B system, cells traversing the reprogramming route can become stabilized in a CD24^high^/SSEA1+ state, which is shown here to correspond to recently discovery transgene-dependent F-class iPSCs, derived from high transgene reprogramming^9^. Cells that are able to acquire a transgene-independent ESC-like iPSC state are characterized by CD24^low^/SSEA1+ expression. Following transgene removal, CD24 also shows the ability to distinguish primed and naive transgene-independent pluripotent states. Published RNA-Seq data comparing mouse ESC and EpiSC states suggest that EpiSCs exhibit higher CD24 levels^45^. Here we have confirmed that CD24 shows strong correlation with the known EpiSC marker, CD40 (ref. 24), and is able to separate ESCs and EpiSCs. Thus, we have shown that three observed states in pluripotent culture could be identified as a function of culture conditions and CD24 expression in the following manner: CD24H cells in DOXH culture, which are transgene-dependent, F-class, iPSCs; CD24L cells in DOX−, which are ESC-like iPSCs; and CD24^high^ cells in DOX−, which are EpiSCs. The dual role of CD24 in delineating these three populations is outlined in Fig. 5b. An important methodological advantage of CD24 during reprogramming is its ability to replace the use of reprogramming state reporters. Other end-stage iPSC surface markers, such as Pecam1 and E-cadherin, have the added difficulty of being sensitive to trypsinization^46^,^47^ and therefore are not ideal for use. Furthermore, CD24 allows for the identification of cells diverting to a transgene-dependent iPSC state, and the separation of this cell type from more ESC-like iPSCs.

Importantly, CD24 also shows conservation in the human system. Consistent with the hypothesis that hESCs are in a primed pluripotent state^30^, they exhibit high levels of CD24, analogous to mouse EpiSCs. Thus, CD24 may provide some utility in tracking the conversion towards a human iPSC state, which is characterized by high CD24 levels^28^,^29^. The recent identification of culture conditions that promote conversion to naive-like hESCs^11^,^48^ enabled the demonstration that CD24 tracks these changes in the human system. Interestingly, during *in vivo* human embryonic development, CD24 levels increase at the single-cell level as the blastocyst undergoes implantation and reaches an epiblast stage^31^. Furthermore, naive-induced hESC populations that were sorted for CD24^low^ cells were further enriched for naive hESC gene expression. Thus, CD24 serves as a marker for resolving heterogeneity in the naive hESC pool.

## Methods

### Identification of cell surface proteins

A streamlined version of the cell surface capture protocol introduced in ref. 12 with simplified sample handling was applied to identify N-glycosylated surface proteins over the project time course. The steps involved are described in detail here. Between 5 × 10^7^ and 30 × 10^7^ cells (individual counts varied by time points) were collected in a 50-ml tube and pelleted through centrifugation (350*g*), and then placed on ice. The pellets were resuspended once in ice-cold labelling buffer (PBS-adjusted to pH 6.5 with 85% phosphoric acid), and then again pelleted through centrifugation.

The cells were subsequently resuspended and oxidized for 15 min in the dark at 4 °C with 1.6 mM sodium-meta-periodate (Sigma) in labelling buffer while being gently agitated. The cell pellet was washed once with labelling buffer to remove residual sodium-meta-periodate and to deplete dead cells and cell fragments. Following centrifugation, the cell pellet was resuspended and the cells exposed to the labelling agent, biocytin hydrazide (Biotium), 5 mM in 10 ml labelling buffer, for 60 min at 4 °C on a rotator on slow speed. On labelling, the cell pellet was washed once with ice-cold labelling buffer to remove unreacted biocytin hydrazide and to deplete dead cells/fragments.

The cell pellet was resuspended in 1 ml of ice-cold 0.1 M ammonium bicarbonate (Sigma) and then transferred to a 1.5-ml Eppendorf tube. After 30 s of indirection sonication on a VialTweeter (Hielscher) at 100% amplitude and 0.5 cycle time, the suspension was centrifuged at 400*g* at 4 °C for 2 min and the size of the (now reduced) pellet noted. Additional sessions of 15 s of sonication followed by centrifugation were performed until the pellets no longer appeared to change in size. The lysate was then centrifuged at 2,500*g* at 4 °C for 15 min. The still-cloudy supernatant was transferred to another Eppendorf tube. After thorough mixing, two 2 l aliquots were set aside to perform a duplicate DC protein assay (Bio-Rad). The remaining supernatant was stored at −80 °C.

The supernatant was thawed on ice once needed. A proportion calculated to hold 5 mg of protein was transferred to a separate tube and the remainder put back in storage. Sufficient 100 mM ammonium bicarbonate was added to bring the volume up to 1 ml, following which 100 ml of 1% RapiGest (Waters) in 100 mM ammonium bicarbonate was added to bring the final concentration to 0.1%. This membrane preparation was indirectly sonicated at 100% amplitude and 0.5 cycle time for 3 min in a VialTweeter to obtain a translucent solution. *Reduction*: 100 mM TCEP (Sigma) in 100 mM ammonium bicarbonate was added to a final concentration of 5 mM TCEP. After 3 min as before in a VialTweeter, the sample was allowed to stand for 30 min at room temperature. *Alkylation*: 500 mM iodoacetamide (Sigma) in 100 mM ammonium bicarbonate was added to a final concentration of 10 mM iodoacetamide and the sample incubated for 30 min in the dark at room temperature. *Digestion*: 50 mg of trypsin (Promega) was added to establish a 1:100 ratio with the measured protein content. The proteins were digested overnight at 37 °C while the sample was gently tumbled. After digestion, the peptide mixture was centrifuged for 10 min at 15,000*g* and the supernatant transferred to a new Eppendorf tube, which was then heated for 15 min at 95 °C to inactivate the trypsin.

UltraLink Streptavidin Plus bead slurry (200 ml; Piercenet) was placed in a Mobicol (Bocascientific) and washed four times with 100 mM ammonium bicarbonate. The beads were resuspended in 100 mM ammonium bicarbonate and added to the peptide mixture. Together, they were incubated for 1 h at room temperature to allow the labelled peptides to bind to the beads. The beads were then returned to a Mobicol and washed intensively: 20 times with 5 M NaCl, 20 times with Stim-Lys buffer, and then finally 20 times with 100 mM ammonium bicarbonate heated to 60 °C. The Stim-Lys buffer consisted of 137 mM NaCl, 50 mM Tris adjusted to pH 7.8 with HCl, 100 mM glycerol, 0.5 mM EDTA adjusted to pH 8.0 with HCl and 1% Triton X (all Sigma). Washing was performed by immersing beads while in the Mobicol, and then spinning out the liquid content by centrifuging for 2 min at 2,000*g*.

The beads were again immersed, in 400 ml of 100 mM ammonium bicarbonate (still within the Mobicol), to which 1 ml of PNGaseF (NEB) was added. They were then gently tumbled overnight at 37 °C. After incubation, the Mobicol was centrifuged for 2 min at 2,000*g* and the eluate collected in an Eppendorf tube. Overall, 500 ml of 100 mM of ammonium bicarbonate in 4% acetonitrile was added to resuspend the beads and was collected in the same manner. The two eluates were combined and then acidified with 150 ml of 10% formic acid to below pH 3.0. This glycopeptide mixture was vortexed until no more bubble formation was apparent, and then stored at −80 °C for later liquid chromatography-mass spectrometry (LC-MS).

Microcolumns were created from capillary-scale nanoflow 75-μm I.D. fused silica tubing (Polymicro Technologies) and then pulled to a fine tip using a P-2000 laser puller (Sutter Instruments). Each of them were packed to a length of 10 cm with 5 μm Luna C18 resin (Phenomenix) using a pressure vessel, and then flushed for 15 min with methanol.

Chromatography buffer ‘A' consisted of 5% acetonitrile and 0.1% formic acid in HPLC-grade water (Fisher), while chromatography buffer ‘B' consisted of 95% acetonitrile and 0.1% formic acid in HPLC-grade water.

Beginning with a proportion of the glycopeptide mixture calculated to be derived from a volume of pre-digestion sample holding 2 mg of total protein, vacuum centrifugation was performed until the volume was concentrated to several ml. The volume was then adjusted to 11 ml with 0.1% formic acid and placed in a well of a 96-well plate, which in turn was placed in an EASY-nLC nano LC pump (Proxeon) connected to a microcolumn.

Microcolumns were regenerated with buffer ‘A' before loading of sample by the nano LC pump. Each chromatography session began with a linear gradient elution of 5–25% buffer ‘B' over 45 min followed by a linear gradient of 25–80% buffer ‘B' over 9 min. A flow rate of 300 nl min^−1^ was maintained. Peptides were analysed using nanospray ionization on an Orbitrap-Velos mass spectrometer (Thermo). MS and MS/MS spectra were acquired with the instrument operating in the data-dependent mode of one MS scan (on the Orbitrap) followed by up to 10 MS/MS scans (on the LTQ-Velos) when triggered by ion signals above a specified threshold. Fragmentation was accomplished using collision-induced association. Three LC-MS replicates were performed for each of the selected time points.

### Database searching and analysis

All MS/MS spectra were searched against the International Protein Index mouse database (Version 3.84) using the SEQUEST algorithm. Confidences in peptide identifications were evaluated using the Statquest probabilistic model^49^ and further filtered to within a mass tolerance of 20 parts-per-million using the accurate ion masses generated by the Orbitrap. This achieved an estimated false-positive rate of 2%. Any identified peptides were then excluded if they did not include the N-glycosylation consensus sequon NxS/T or did not demonstrate the asparagine to aspartic acid deamidation of 0.986 Da resulting from the treatment with PNGaseF. Relative quantities of cell surface proteins were assessed using spectral counting. The mass spectrometry proteomics data have been deposited to the ProteomeXchange Consortium ( http://proteomecentral.proteomeexchange.org) via the PRIDE partner repository with the data set identifier PXD001456.

### Culture and reprogramming of pluripotent mouse cells

MEFs (inducible 1B lines^7^ and Col1a1 lines^21^) and control mouse ES cells (R1 line) were maintained in Dulbecco's modified Eagle medium (DMEM) supplemented with 10% (v/v) fetal bovine serum (FBS; Gibco), 1 mM sodium pyruvate (Gibco), 2 mM Glutamax (Invitrogen) and 1% (v/v) penicillin and streptomycin (Invitrogen). Mouse fibroblasts were reprogrammed in serum-containing mouse ESC medium consisting of DMEM supplemented with 15% (v/v) FBS (Wisent), 0.1 mM β-mercaptoethanol (BME, Sigma), 1 mM sodium pyruvate, 0.1 mM MEM nonessential amino acid (NEAA, Gibco), 2 mM Glutamax, 1% (v/v) penicillin–streptomycin and 1,000 U ml^−1^ of LIF (Millipore). EpiSCs (CDE) used for control staining were cultured as previously described^25^.

For reprogramming, secondary MEF 1B cells (isolated as previously described^7^ from tetraploid complementation and chimeras) or MEF Col1a1 cells^21^ were seeded as reported^10^, in 0.2% gelatin-coated T25 or T75 plates (Sarstedt). Cells were maintained in DOX (Sigma) high culture (1.5 μg ml^−1^) and were fed regularly and passaged at sampling time points and whenever confluence was reached. Cells were maintained on three DOX treatment time courses: DOX-high (DOXH), DOX-low-to-negative (DOXL−) and DOX-high-to-negative (DOXH−) time course, as previously described^10^. For the DOXH time course, cells were maintained in DOX-high culture (1.5 μg ml^−1^). For the DOXL− time course, cells were passaged on day 8 and half of the cells were transitioned to a DOX-low level (either 5 ng ml^−1^). DOX-low-treated cells subsequently underwent passaging and DOX removal on day 14. For the DOXH− time course, DOX-high cells were transitioned to DOX− culture.

For derivation of EpiSCs from iPSCs and ESC controls, cells were maintained either in standard ESC culture (serum+LIF), 2i culture supplemented with 3 μM CHIR99021 and 1 μM PD0325901 (Reagents Direct), or standard EpiSC culture, as previously described^25^. Following 18 days of culture in these conditions, with regular passaging, the cells were sampled for FACS and qPCR analyses.

Cells were incubated in a humidified 5% (v/v) CO_2_ air environment at 37 °C.

### Flow cytometry and immunocytochemistry staining

Antibodies used for flow cytometry are listed in Supplementary Table 1. Surface stainings for flow cytometry were performed in the presence of 7AAD (Molecular Probes) or LIVE/DEAD Fixable Near-IR Dead Cell Stain (Life Technologies), and populations were gated on live cells. Flow cytometry was conducted on an LSRFortessa (BD Biosciences). For cell sorting, cells labelled with antibodies above and sorted using a FACSAria flow cytometer (BD Biosciences) or MoFlo Astrios (Beckman Coulter). Analysis was performed on FACSDiva (BD Biosciences) as well as FlowJo (Tree Star).

Immunocytochemistry stainings were performed by fixing cells in PBS containing 4% (v/v) formaldehyde. Cells were permeabilized in PBS containing 0.1% (v/v) Triton X-100 and subsequently blocked in PBS containing 10% (v/v) donkey serum. Samples were incubated with primary and secondary antibodies in PBS containing 1% (w/v) BSA and imaged using a confocal microscope (FV1000 laser scanning confocal; Olympus). Images represent the z-stack projection of confocal optical sections. EdU cell proliferation assays were performed according to the manufacturer's protocol using the Click-iT EdU Pacific Blue Flow Cytometry Assay Kit (Invitrogen).

### Quantitative PCR analysis

Total RNA was extracted from cells using Qiagen RNAeasy miniprep columns according to the manufacturer's protocol. Total RNA was used to generate cDNA using Superscript III reverse transcriptase (Invitrogen) according to the manufacturer's instructions. Generated cDNA was mixed with respective primers and SYBR green mix (Roche, Sigma) and run on an Applied Biosystems 7900 HAT real-time PCR machine. Relative expression of described genes was determined by the delta–delta cycle threshold (*C*_t_) method with the expression of *Gapdh* (or *GAPDH*) as an internal reference. Primer sequences are listed in Supplementary Table 2.

### Differentiation protocols

Differentiation was conducted as previously reported^19^. Mesoderm and endoderm differentiation was carried out by dissociating iPSC and seeding in low-adhesion plates at a density of 1 × 10^6^ cells per 10 ml in DMEM containing 15% (v/v) FBS, 1% (v/v) penicillin and streptomycin, 2 mM Glutamax, 0.1 mM BME and 0.1 mM NEAA. Cells were cultured for 4 days on an orbital shaker (65 r.p.m.) with medium exchange on day 2. After 4 days, suspension aggregates were seeded on gelatin-coated tissue-culture plates and cultured for another 5 days before staining with antibodies.

Ectoderm differentiation was performed by trypsinizing and plating iPSCs at 5 × 10^5^ cells per 10 ml in SFEB medium (Glasgow minimum essential medium supplemented with 5% (v/v) knockout serum replacement, 0.1 mM NEAA, 1 mM sodium pyruvate, 1% (v/v) penicillin and streptomycin and 0.1 mM BME). Cells were cultured for 3 days in low-adherence plates. On day 3, cells were re-fed by replacing 70% of the medium and were cultured for another 2 days. Spheres were transferred intact to Geltrex-coated six-well plates and incubated for 5 days in N2B27 medium (DMEM with F12 and Neurobasal medium supplemented with B27, N2 supplements, 0.005% (w/v) BSA and 1 mM sodium pyruvate) before being stained with antibodies.

### Human ESC cultures and induction to naive state

H9 hESCs were obtained from the WiCell Research Institute. HES2 hESCs were provided by G. Keller (McEwen Centre for Regenerative Medicine/University Health Network). HES2 and H9 cells were cultured on Geltrex LDEV-Free Reduced Growth Factor Basement Membrane Matrix (Life Technologies)-coated plates in Nutristem hESC XF Culture Media (Biological Industries), supplemented with 1 × Penicillin–Streptomycin (Life Technologies). H9 cells were passaged 1:12–1:24 every 5–6 days and were disassociated into small clumps using 0.1% collagenase IV (Invitrogen). HES2 cells were passaged 1:12–1:24 every 5-6 days and were dissociated to single cells using TrypLE Express (Life Technologies). All cell-line stocks were confirmed negative for mycoplasma contamination.

To convert H9 and HES2 hESCs to a naive state, primed hESCs were passaged on feeder layers of irradiated mouse embryonic fibroblasts in NHSM media as described previously^11^ with modifications recommended by J. Hanna. In brief, NHSM media contained Knockout DMEM (Life Technologies), 15% Knockout Serum Replacement (Life Technologies), 1 × glutamax (Life Technologies), 1 × NEAAs (Life Technologies), 1 × penicillin–streptomycin (Life Technologies). Added to this base media were 12.5 μg ml^−1^ Insulin (Sigma), 20 ng ml^−1^ human Leukemia Inhibitory Factor (made in-house^11^), 20 ng ml^−1^ human LR3-IGF1 (prospec), 2 ng ml^−1^ tgfb1 (RnD), 12 ng ml^−1^ fgf2 (peprotech), 1 μM PD0325901 (Reagents Direct), 3 μM CHIR99021 (Reagents Direct), 5 μM SP600125 (Santa Cruz Biotechnology), 2 μM BIRB796 (Cayman Chemical), 5 μM Y-27632 (Reagents Direct) and 0.4 μM LDN193189 (Cayman Chemical). Subsequent experiments involving naive induction were conducted with an altered induction cocktail in which LDN and IGF1 were removed and the following reagents were added: 0.25 mg ml^−1^ Albumax (Invitrogen), 1 × N2 supplement (Life Technologies), 500 μg ml^−1^ Ascorbic acid (Sigma) and 1 μM Go6983 (Tocris Biosciences). HES2 cells were passaged 1:30 every 4–5 days, and H9 cells were passaged 1:30 every 5–6 days on irradiated MEF-coated plates and were dissociated into single cells using TrypLE Express (Life Technologies).

### Statistical and data analysis

Results were expressed as mean±s.d. from replicates indicated in figure legends. Statistical tests were conducted as reported for each figure. A minimum sample replicate size of *n*=3 was used for all data analyses to allow for appropriate statistical testing. Calculations for statistical significance were performed using Excel and MATLAB software. For most samples, a Student's *t*-test was used for pairwise comparison of an experimental condition of interest compared to control. Hierarchical clustering of gene expression data was conducted using the clustergram command in MATLAB. PCA and K-means clustering analysis of surface proteome data in reprogramming time course was performed using R.

## Additional information

**Accession codes**: The mass spectrometry proteomics data have been deposited to the ProteomeXchange Consortium (http://proteomecentral.proteomeexchange.org) via the PRIDE partner repository with the data set identifier PXD001456.

**How to cite this article:** Shakiba, N. *et al.* CD24 tracks divergent pluripotent states in mouse and human cells. *Nat. Commun.* 6:7329 doi: 10.1038/ncomms8329 (2015).

## Supplementary Material

Supplementary Information Supplementary Figures 1-16 and Supplementary Tables 1-2

Supplementary Data 1 Deconvolution of cell surface protein isoforms. A single gene can yield different protein products, through alternate splicing and other means. These different proteins (isoforms) share a portion of their amino acid sequences. Peptides derived from shared portions cannot be attributed to a particular isoform. As a result, protein isoform groups have been quantified as a collective under a single gene and primary protein ID. However, a record of the isoforms potentially mapped has been kept and is presented here.

Supplementary Data 2 N-glycosylated peptides. The list of all peptides that (1) were derived from DOX high time course samples prepared using the cell surface capture protocol, (2) passed the STATQUEST identification confidence filter, (3) had observed masses within 20 parts-per-million of the masses predicted for their sequences, and (4) contain at least the start of a N-glycosylation sequon ('NxS' or 'NxT', where x is any amino acid save proline) in their sequence.

Supplementary Data 3 Percentage proportions of cell certain proteins by GO biological category across the DOX high time course. A Panther (ver 8.0) gene list analysis was performed for genes corresponding to the set of cell surface proteins identified for each point of the Project Grandiose time course. The functional classifications were tallied and tabulated as a relative percentage of each list. The proportion of each classification was similar across the whole time course, except that 'catalytic activity' slightly overtook 'receptor activity' on days 16 and 18.

Supplementary Data 4 Raw, normalized, and averaged spectral counts. The cell surface capture data. 1. Spectral counts have been tallied for the different peptides of a given proteoform (protein isoform) group for each mass spectrometry replicate. 2. Since a fixed amount of protein was used for each injection, it might reasonably be expected that the number of spectral counts observed should be similar across samples. Spectral counts were normalized for a replicate by dividing each by the square root of (400 over the total observed for that replicate).The square root was used as a balance between the expectation of similar counts and the possibility of a real difference. 3. Finally, the normalized spectral counts were averaged across the replicates to produce a single value per time point. The absence of spectral counts was treated as a value of zero for the purpose of averaging.

Supplementary Data 5 Combined cell surface protein data and associated global proteome data. A list of those proteins of the project's global proteomics data which overlapped those proteins identified by cell surface capture. The quantitative values are relative, not absolute, and are on a log 2 scale. There is no basis for comparison between proteins, only between time points for a given protein. For example, if protein A has a value of 0.0 on Day 0, and protein B has a value of -1.0, that does not indicate there was twice of much of protein A as protein B on that day.

Supplementary Data 6 Numbers of GO biological categories in which proteins were over-represented (p < 0.05). Panther (ver 8.0) gene list analysis was performed for (1) the list of proteins found to be downregulated between days 0 and 2, (2) the subset of surface proteins overlapping this list, and (3) the complementary subset (remainder) of proteins on the list but not among the subset of surface proteins. The reported number of GO biological categories was greater for the subset of surface proteins that for the complementary subset despite the latter's much larger size. This held true for the list of proteins observed to be upregulated from day 18 of the DOX high time course to the final secondary iPSC state, and regardless of whether the cutoff for determining downregulation/upregulation was defined to be two-fold or three-fold: in each instance, the cell surface protein subset accounted for more of the over-represented categories than the remaining subset of the list.
