# Supplementary material for: CD24 tracks divergent pluripotent states in mouse and human cells
Source: Nat Commun. 2015 Jun 16;6:7329. doi: 10.1038/ncomms8329 (PMC4490408; doi:10.1038/ncomms8329)
Supplement: Supplementary Information — Supplementary Figures 1-16 and Supplementary Tables 1-2 [file ncomms8329-s1.pdf]

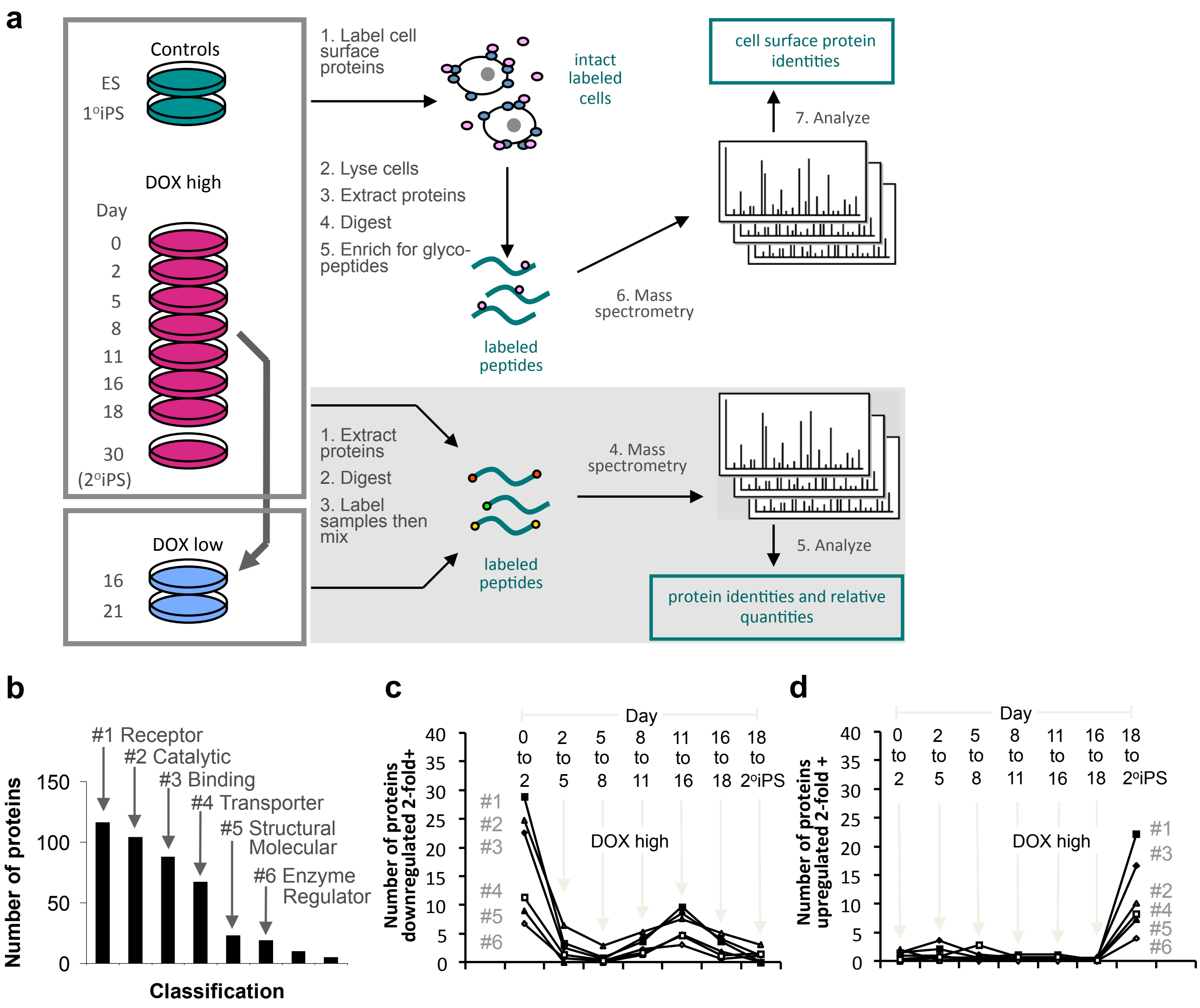

Supplementary Figure 1: Surface proteomics during reprogramming. a, Overview of mass spectrometry analysis, including sample preparation. **b,** Summary of distribution of surface protein classes during reprogramming. Data bars show mean (n=3 biological replicates). **c,** Number of cell surface proteins by functional category (from Supplementary Figure 1a) displaying a 2-fold or greater downregulation over intervals of the DOX-high time course. **d,** Number of cell surface proteins by functional category (from Supplementary Figure 1a) displaying a 2-fold or greater upregulation over intervals of the DOX-high time course.

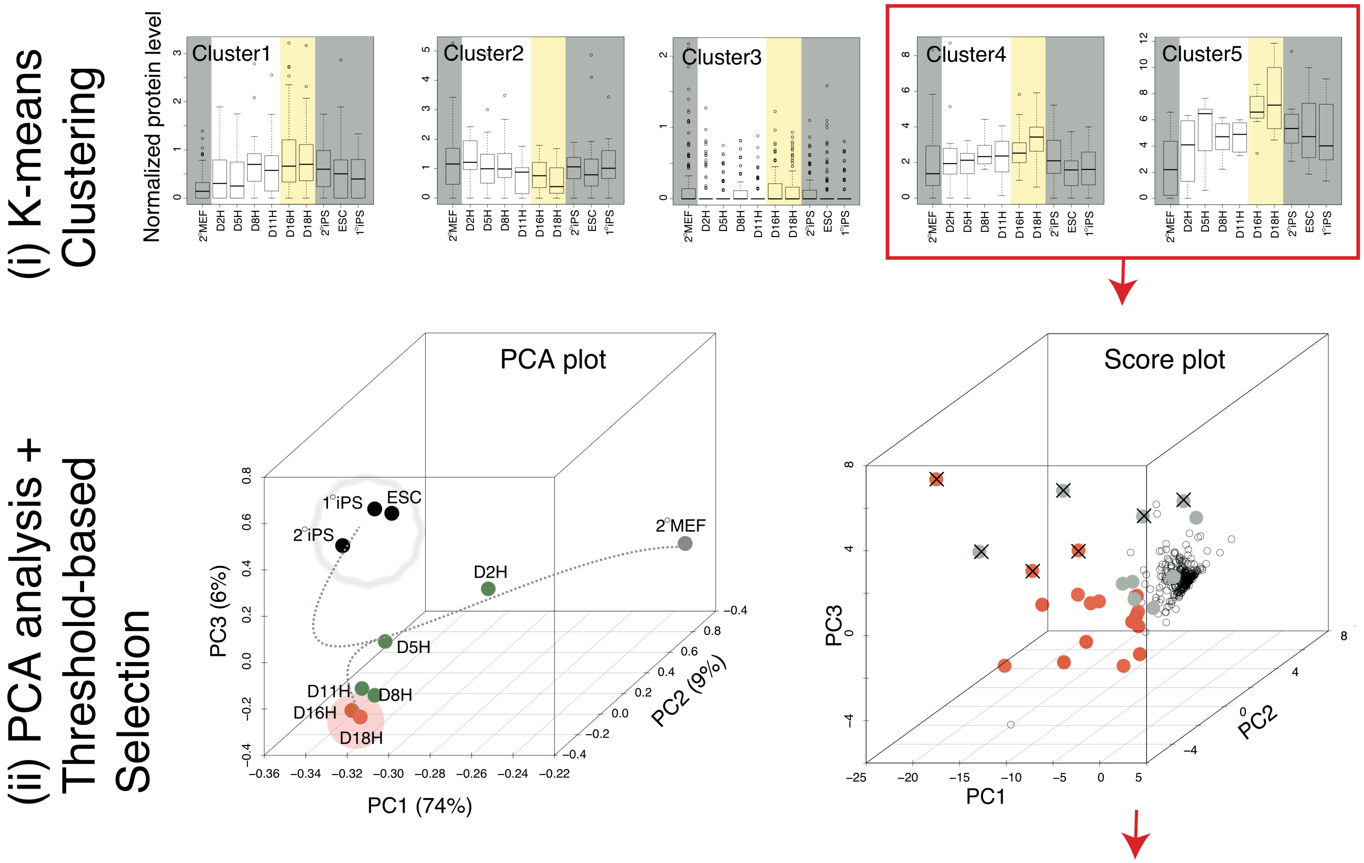

(iii) Candidate proteins

Gene Name	Description	Protein ID (UniProt)
Cadm1	Cell adhesion molecule 1	Q8R5M8
Cd24a	Signal transducer CD24	P24807
Ctse	Envelope polyprotein	P10404
Emp1	Epithelial membrane protein 1	P47801
Lama1	Laminin subunit alpha-1	P19137
Lama5	Laminin subunit alpha-5	Q61001
Lamb1	Laminin subunit beta-1	P02469
Lamc1	Laminin subunit gamma-1	P02468
Lamp1	Lysosome-associated membrane glycoprotein 1	P11438
Scarb2	Lysosome membrane protein 2	O35114
Slc7a1	High affinity cationic amino acid transporter 1	Q09143
Alpl	Alkaline phosphatase: tissue-nonspecific isozyme	P09242
Atp1b3	Sodium/potassium-transporting ATPase subunit beta-3	P97370
Bsg (Cd147)	Basigin	P18572
Nptn	Neuroplastin	P97300

Supplementary Figure 2: Identification of interesting surface protein candidates based on K-means clustering and PCA analysis. Overview of approach used to select surface proteins of interest for subsequent validation, with CD24 emerging as a particularly interesting candidate. Following K-means clustering, genes were selected that were differentially expressed in the F-class state. Principal component analysis of surface proteomics data was conducted using the complete surface proteome dataset including all measurable surface proteins. The contribution of each protein to the PCA components was determined, identifying those that contribute mainly to PCA3 (shown with an X), which is mainly ESC-specific, and those that do not significantly separate the MEF and F-class states, providing less than a two-fold difference between those states (shown in gray). The remaining (orange) candidates are listed in the table, of which CD24 is a promising candidate.

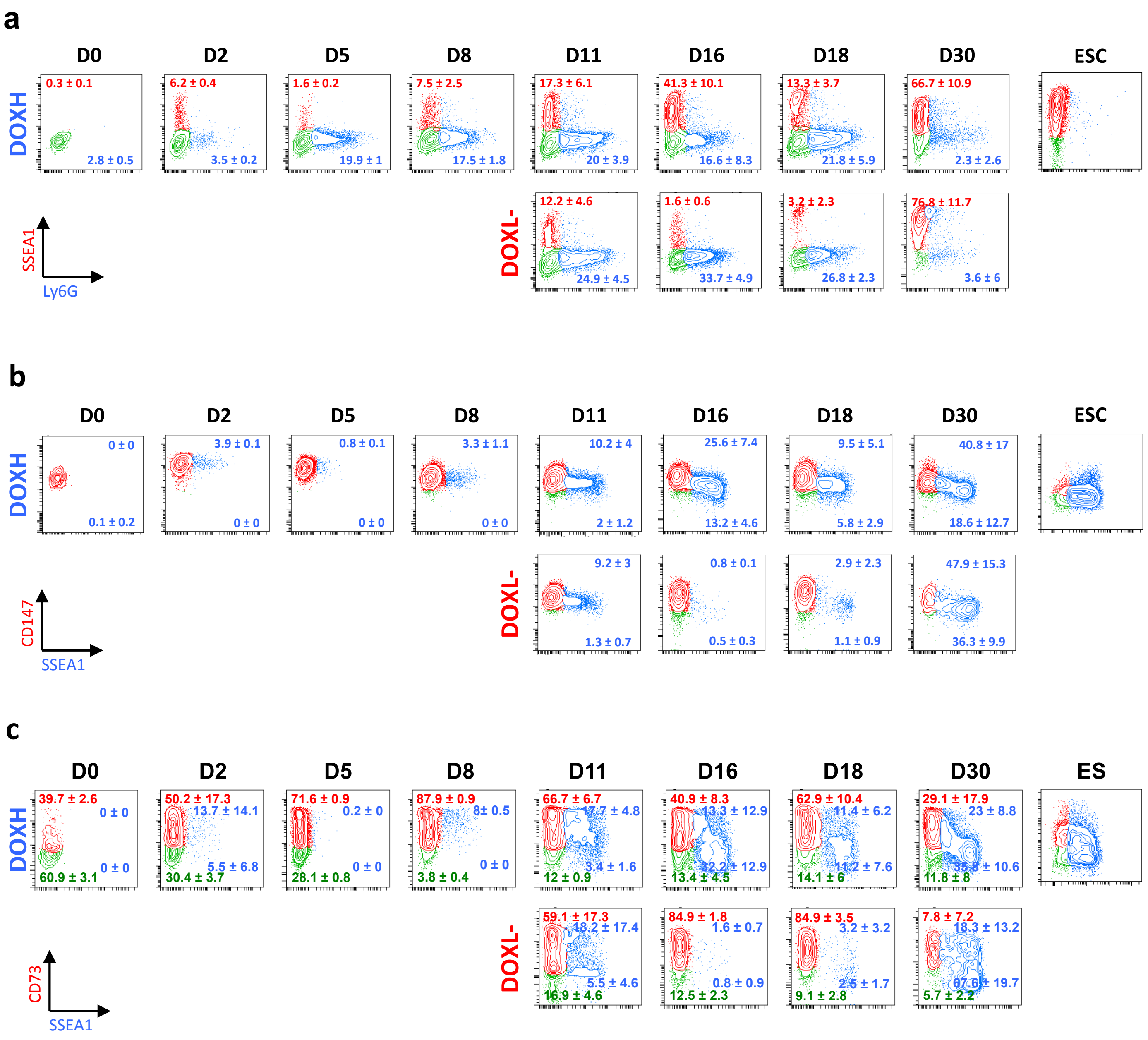

Supplementary Figure 3: Flow cytometry analysis of mass spectrometry hits. a, Flow cytometry analysis of Ly6G/SSEA1 expression during DOXH/DOXL- reprogramming culture. Flow plots are representative from 3 technical replicates. **b,** Flow cytometry analysis of CD147/SSEA1 expression during DOXH/DOXL- reprogramming culture. Flow plots are representative from 3 technical replicates. **c,** Flow cytometry analysis of CD73/SSEA1 expression during DOXH/DOXL- reprogramming culture. Flow plots are representative from 3 technical replicates.

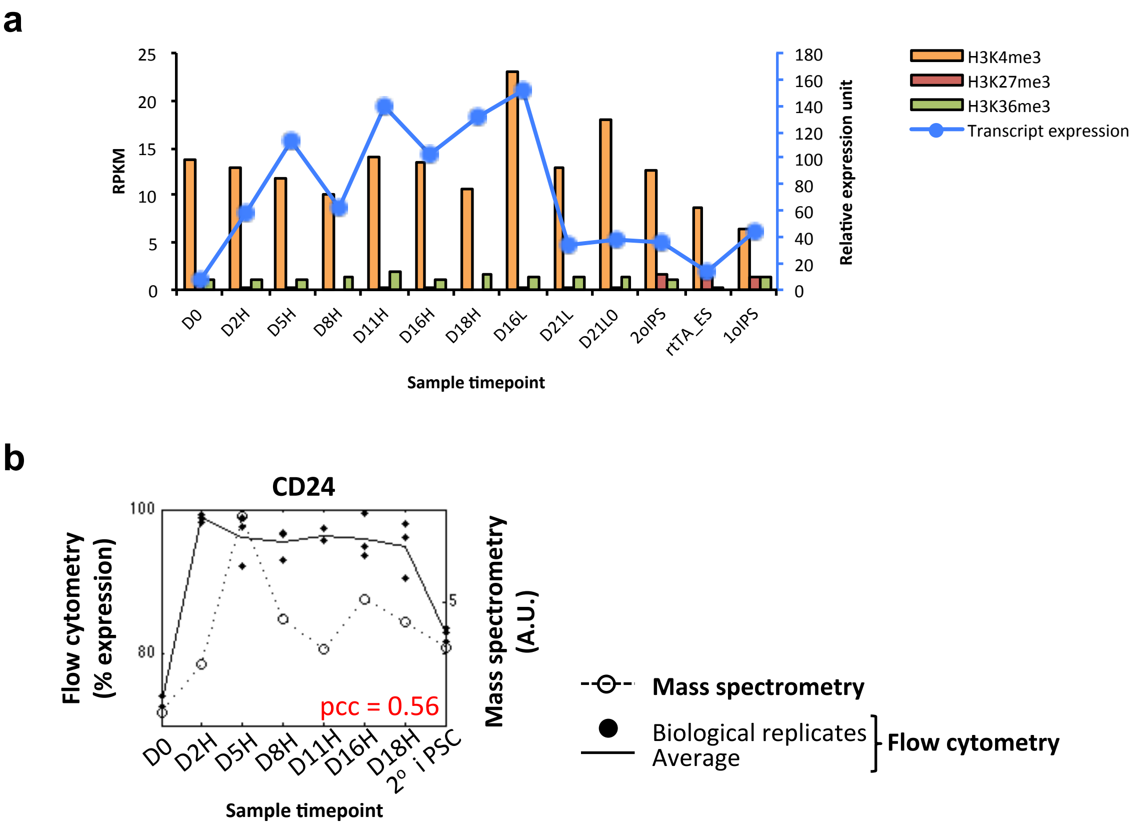

Supplementary Figure 4: Cross-platform CD24 expression time course. a, Summary of transcriptome expression and histone methylation marks in the CD24 locus, collected from Project Grandiose 'omics analysis[10]. **b,** Comparison of CD24 expression levels as found by flow cytometry and mass spectrometry with partial correlation coefficient (pcc) shown. n=3 biological replicates.

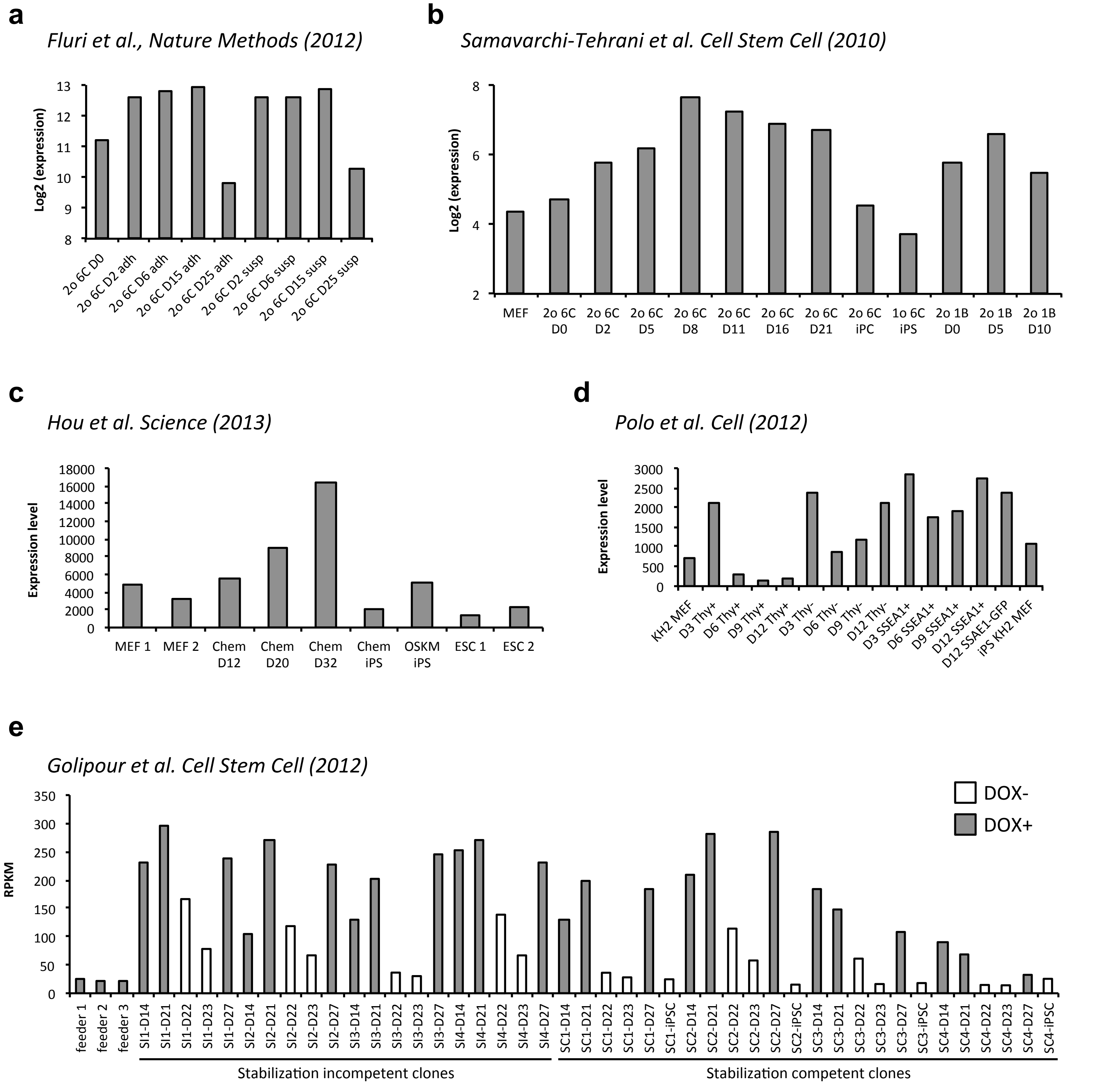

Supplementary Figure 5: CD24 in published reprogramming systems. a, CD24 expression levels in secondary 6C MEF reprogramming in adherent and suspension systems, derived from microarray analysis[19]. **b,** CD24 expression levels in primary and secondary 6C MEF and secondary 1B MEF reprogramming along the reprogramming time course, derived from microarray analysis[3]. **c,** CD24 expression levels in small molecule-induced reprogramming MEFs, derived from microarray analysis[20]. **d,** CD24 expression levels in MEFs, sorted Thy1+/- cells, SSEA1+ cells, and iPS cells throughout reprogramming, derived from microarray analysis[4]. **e,** CD24 expression levels in "stabilization competent" and "incompetent" clones throughout the reprogramming process, derived from RNA-seq analysis[8].

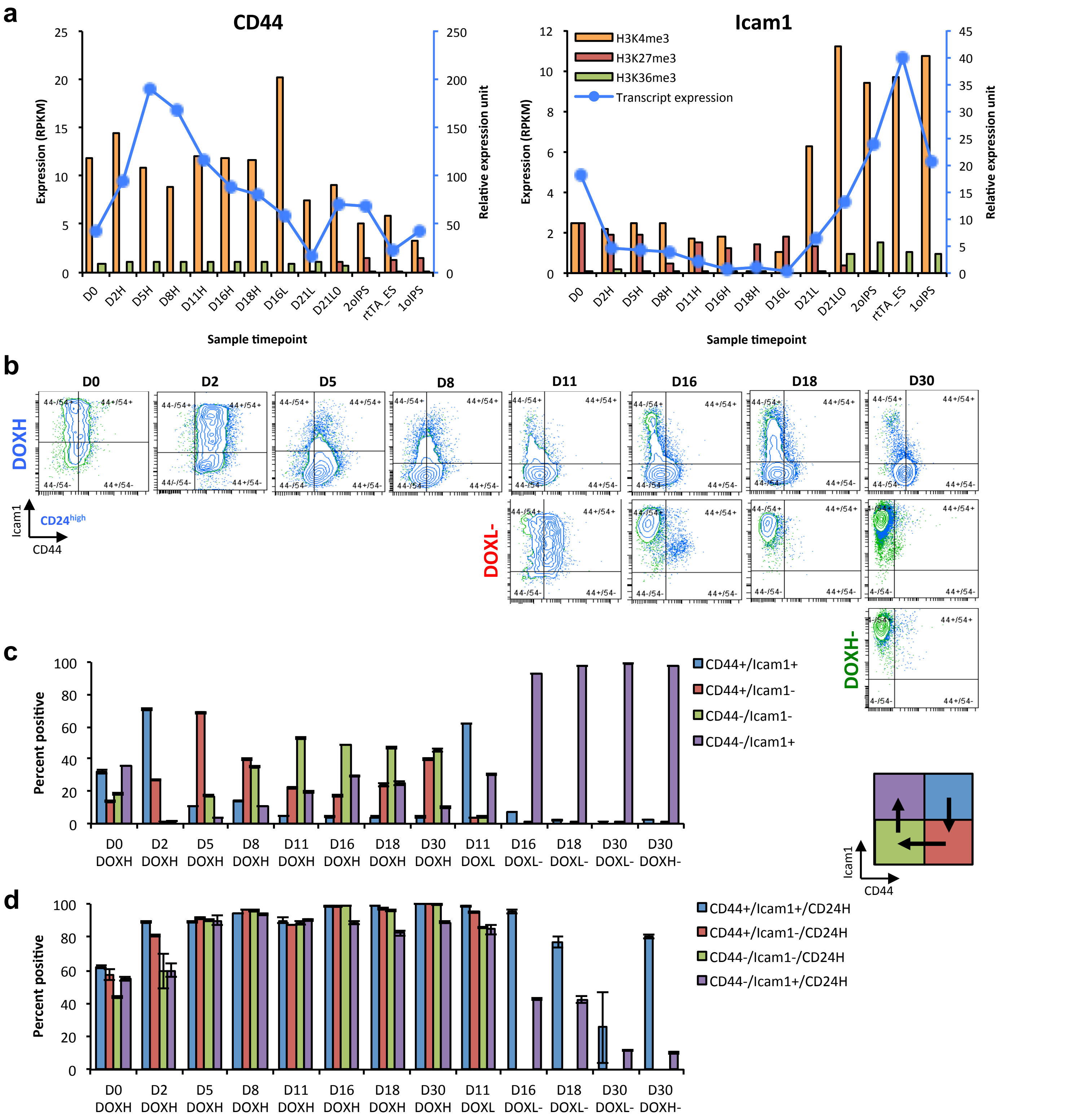

Supplementary Figure 6: Co-staining for CD24/CD44/Icam1 expression in secondary 1B reprogramming time course. a, Transcriptome and epigenetic data taken from Project Grandiose ‘omics analysis[10], showing transcriptome CD44 and Icam1 expression and histone methylation marks at the twp loci. **b,** Flow cytometry analysis of CD24/CD44/Icam1 expression levels during reprogramming of secondary MEF 1B cells derived from tetraploid complementation. CD24^{high} cells are indicated in blue. Flow plots are representative from 3 technical replicates. **c,** Summary of percent CD44/Icam1 levels during reprogramming from flow cytometry analysis. Data bars show mean±s.d. (n=3 technical replicates). **d,** Summary of percent CD24^{high} cells within the CD44/Icam1 subsets from flow cytometry analysis. Data bars show mean±s.d. (n=3 technical replicates).

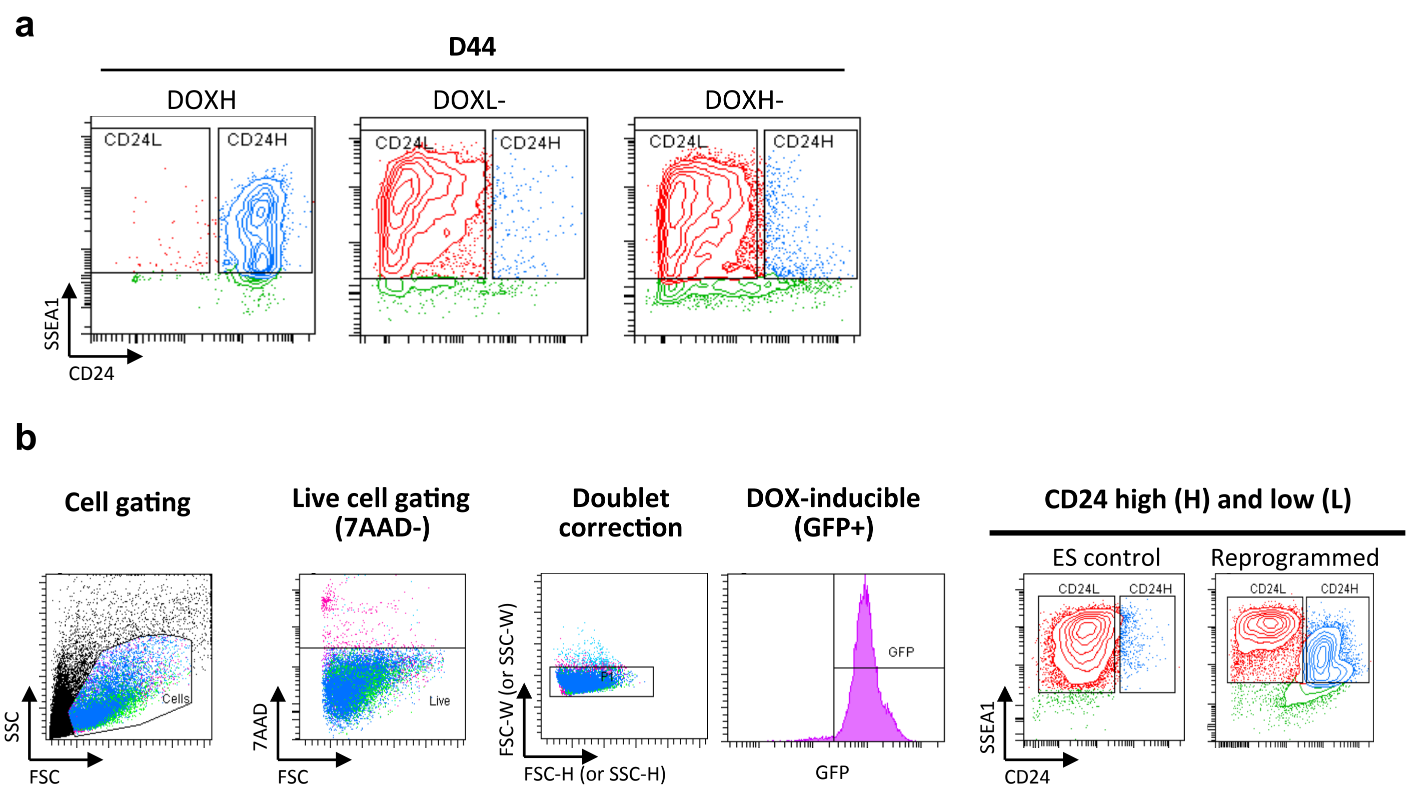

Supplementary Figure 7: Summary of flow cytometry analysis for CD24 expression in secondary reprogramming MEFs. a, CD24/SSEA1 staining of D44 cells from DOXH, DOXL-, and DOXH- time courses. Flow plots are representative of 3 technical replicates **b,** Summary of flow cytometry gating strategy used to quantify the emergence of two divergent reprogramming populations: $CD24^{high}$/SSEA1+ (CD24H) and $CD24^{low}$/SSEA1+ (CD24L).

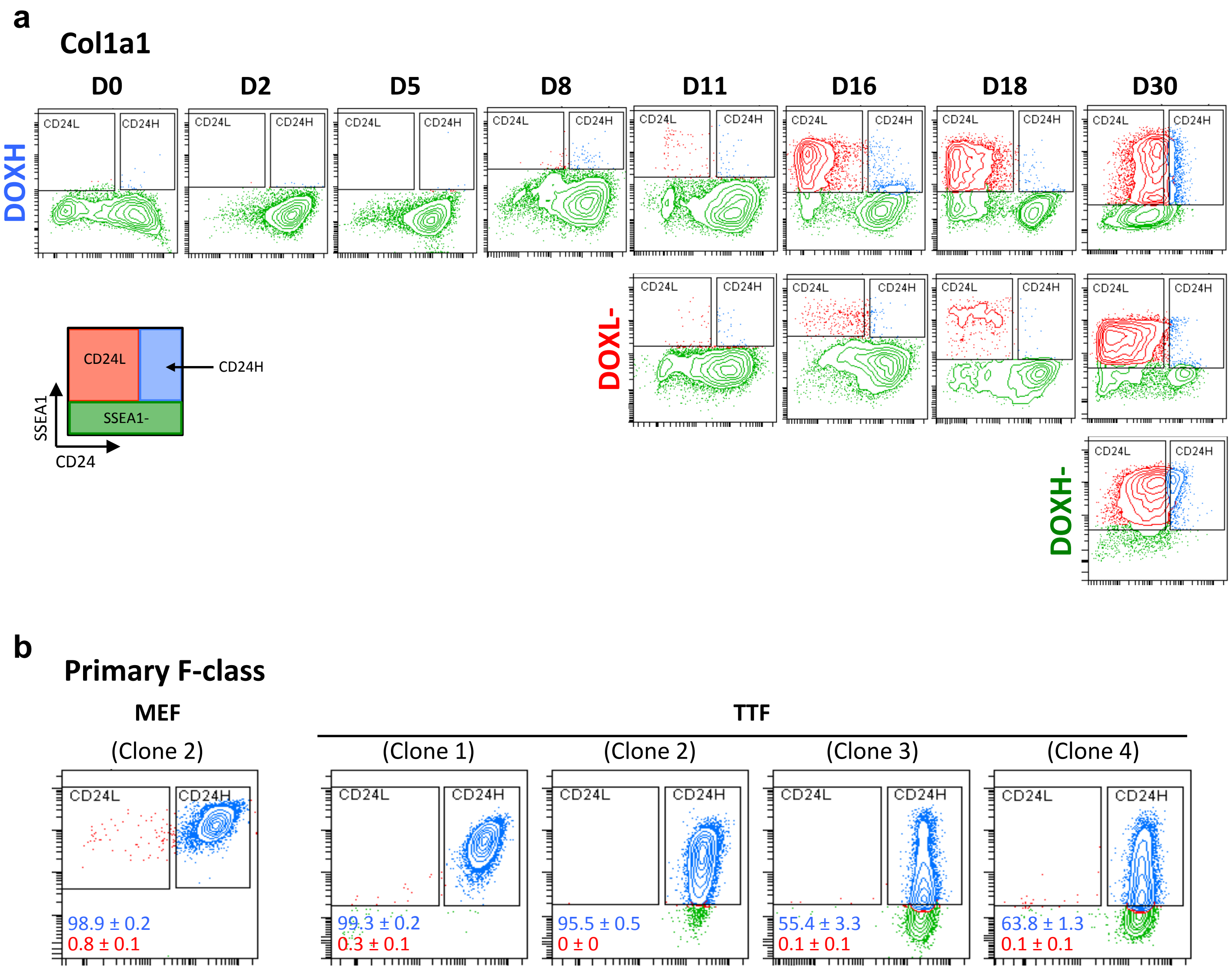

Supplementary Figure 8: CD24/SSEA1 expression levels in a secondary MEF Col1a1 reprogramming system and primary 1B-derived F-class cells. a, CD24/SSEA1 staining of reprogramming time course with DOXH, DOXL-, and DOXH- treatment paths. Flow cytometry plots are representative of 3 technical replicates. **b,** Representative flow cytometry plots of CD24 cs. SSEA1 expression in F-class cells derived from Primary 1B reprogramming (Clone 2 from Tonge *et al.*) and Tail Tip Fibroblasts (Clones 1-4 from Tonge *et al.*)[9]. Flow cytometry plots are representative of 3 technical replicates.

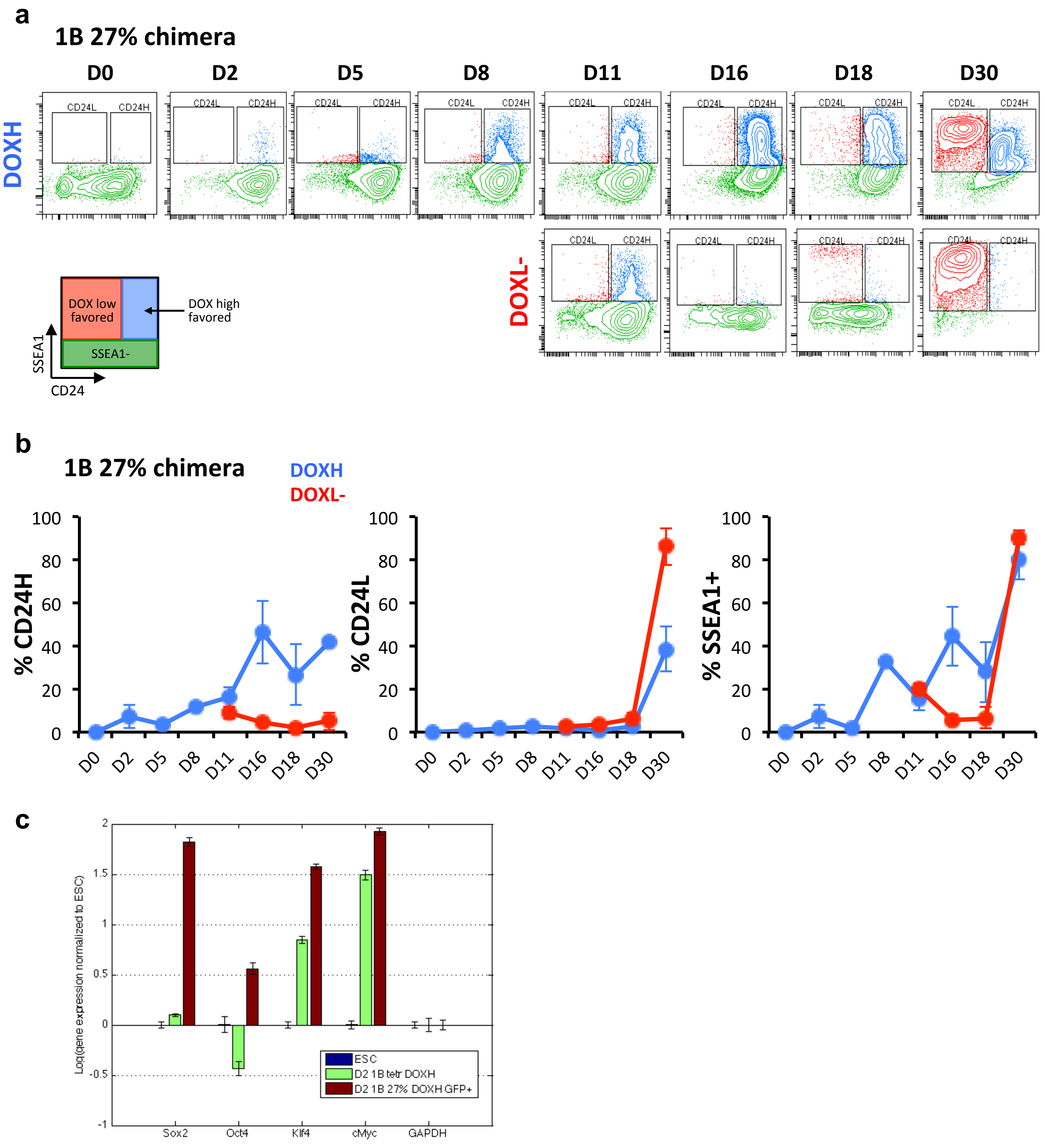

Supplementary Figure 9: Analysis of emerging CD24H and CD24L subpopulations in an alternate secondary MEF 1B reprogramming system. a, Representative flow cytometry plots of CD24/SSEA1 expression during reprogramming of secondary MEF 1B cells derived from a 27% chimera. Flow plots are representative of 3 biological replicates. **b,** Percentage of CD24H/L and SSEA1+ cells in DOXH, DOXL-, and DOXH- culture time courses. Data bars show mean±s.d. (n=3 biological replicates). **c,** OKMS transgene expression levels, normalized to ESC, after 2 days of DOX-induction of various 1B secondary MEFs derived from tetraploid complementation (tetr) and 27% chimeras (cells sorted for GFP+ expression, indicating OKMS transgene availability). Data bars show mean±s.d. (n=3 technical replicates).

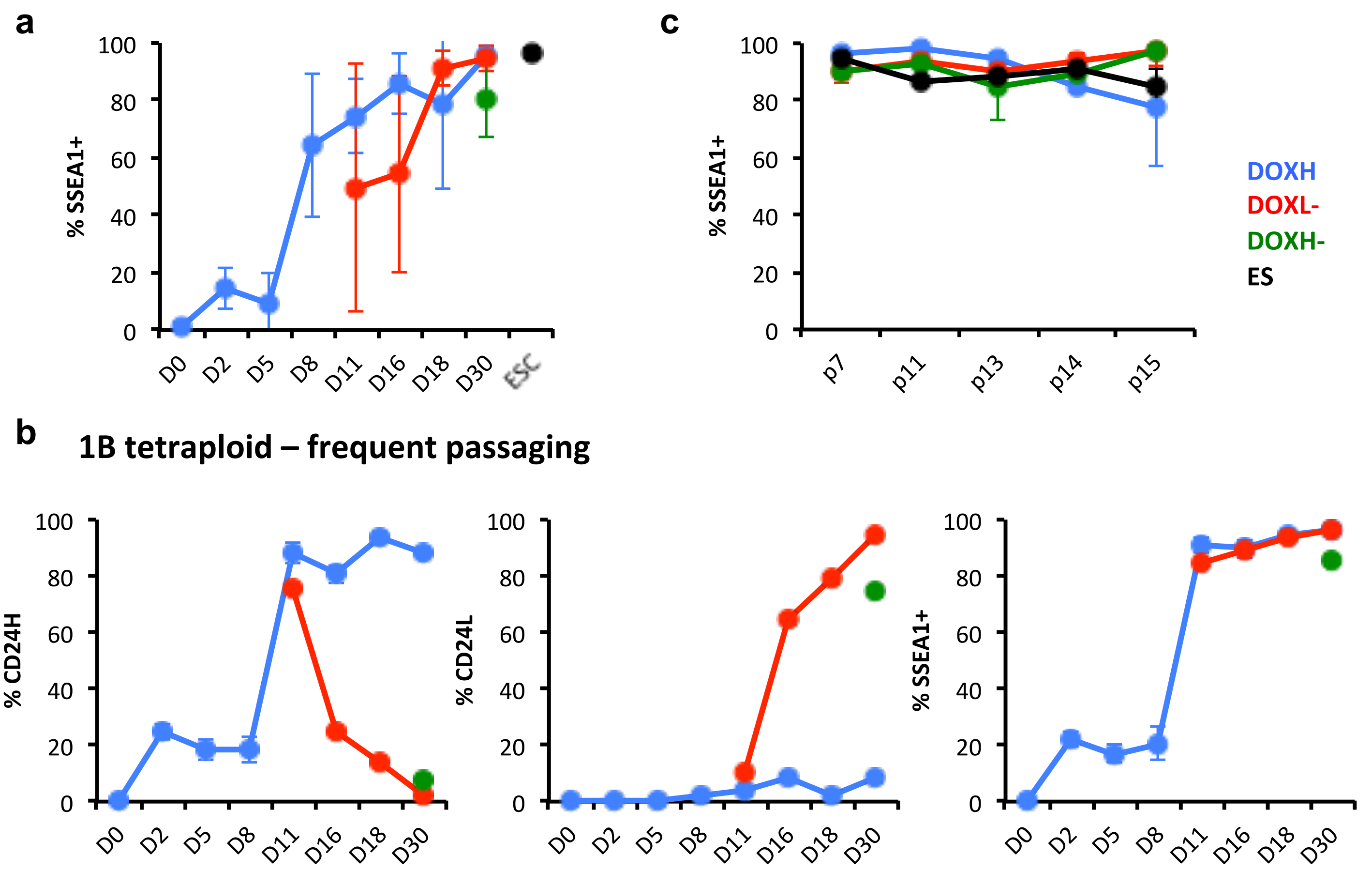

Supplementary Figure 10: CD24/SSEA1 expression levels in a secondary MEF 1B reprogramming system derived from tetraploid complementation. a, Percent SSEA1 expression in DOXH, DOXL-, and DOXH- reprogramming time courses. Data bars show mean±s.d. (n=3 biological replicates). **b,** Effect of frequent passaging (serial sampling of reprogramming cells, requiring passaging at every time point) on the emergence of CD24H and CD24L subpopulations as well as SSEA1 expression in the DOXH, DOXL-, and DOXH- reprogramming time courses. Data bars show mean±s.d. (n=3 technical replicates). **c,** Percent SSEA1 expression in long-term passaged D30-sorted DOXH CD24H, DOXL- CD24L, and DOXH- CD24L cells. ESC cells are included as a control. Data bars show mean±s.d. (n=3 technical replicates).

a

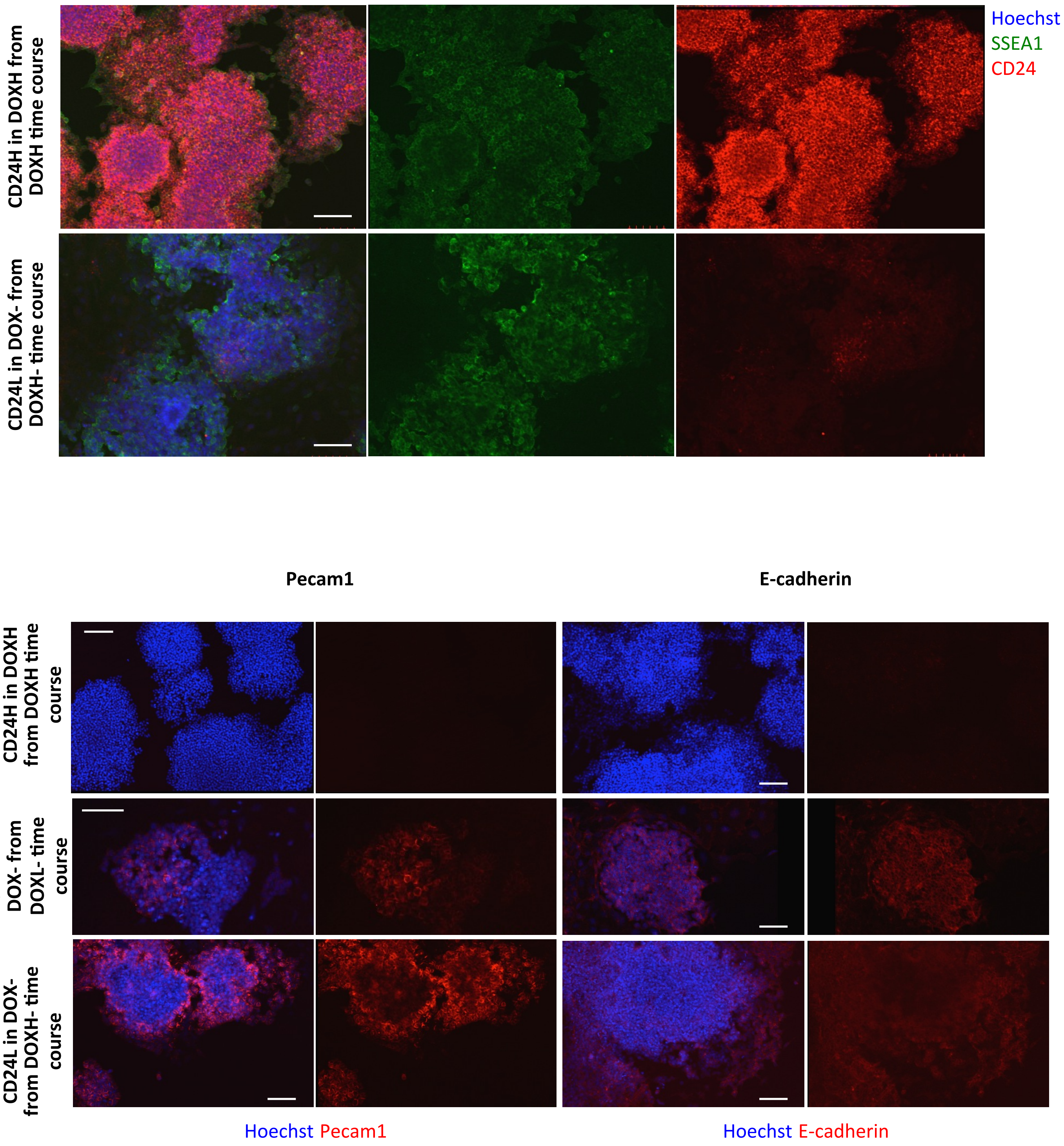

Supplementary Figure 11: Confocal staining of CD24H and CD24L reprogramming cells emerging from DOXH, DOXL-, and DOXH- culture. a, Confocal staining for CD24 and SSEA1 protein expression in CD24H and CD24L cells derived from DOXH and DOXH- time courses. Confocal images are representative of 3 technical replicates. Scale bar represents 100 µm. **b,** Confocal staining for Pecam1 and E-cadherin protein expression in CD24H and CD24L cells derived from the DOXH, DOXL-, and DOXH- time courses. Confocal images are representative of 3 technical replicates. Scale bar represents 100 µm.

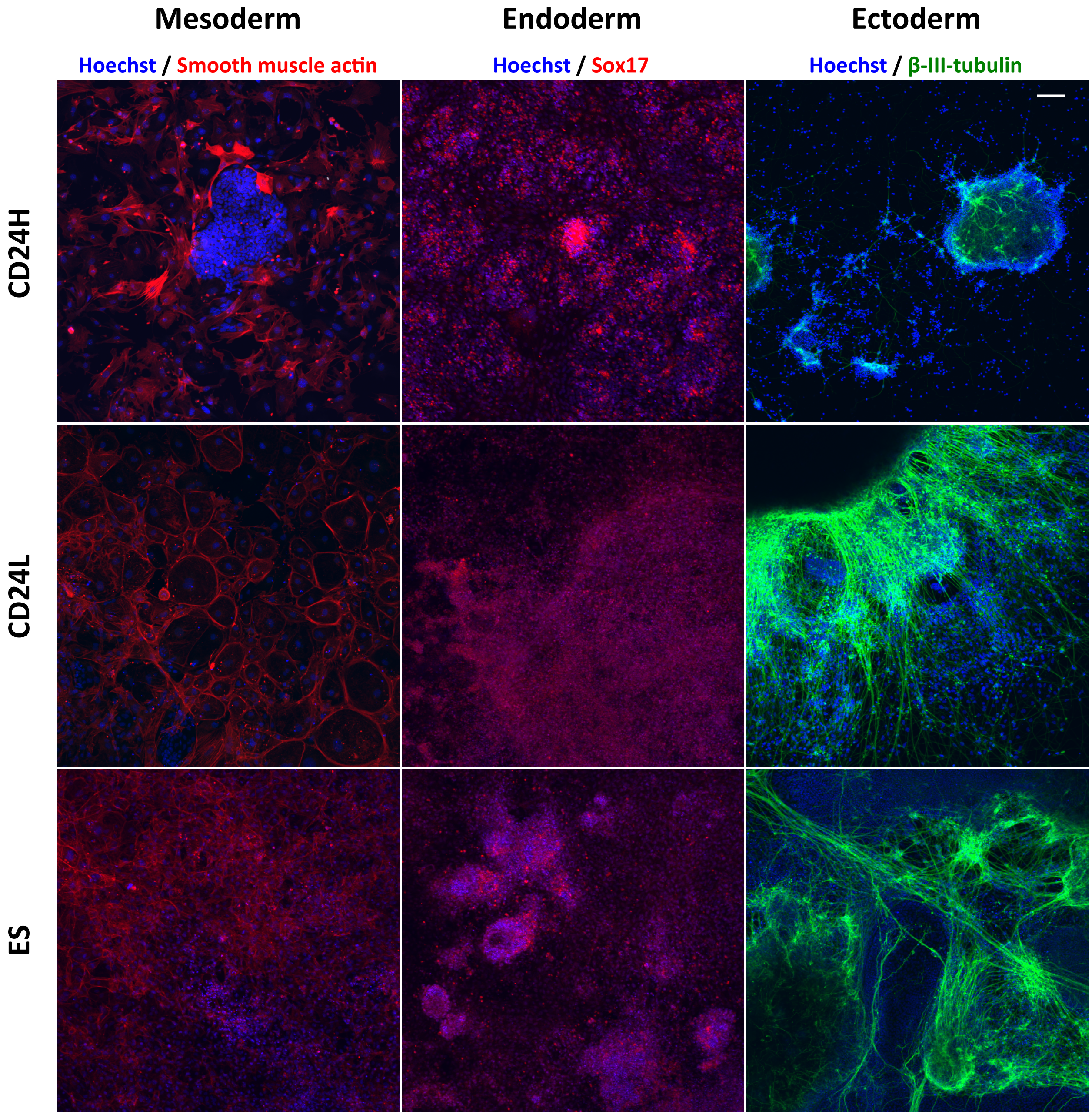

Supplementary Figure 12: Assessment of pluripotency of CD24H and CD24L cells via *in vitro* differentiation. *In vitro* differentiation of CD24H and CD24L cells to mesoderm (smooth muscle actin), endoderm (Sox17), and ectoderm (β-III-tubulin) lineages. Confocal images are representative of 3 technical replicates. Scale bar represents 100 μm.

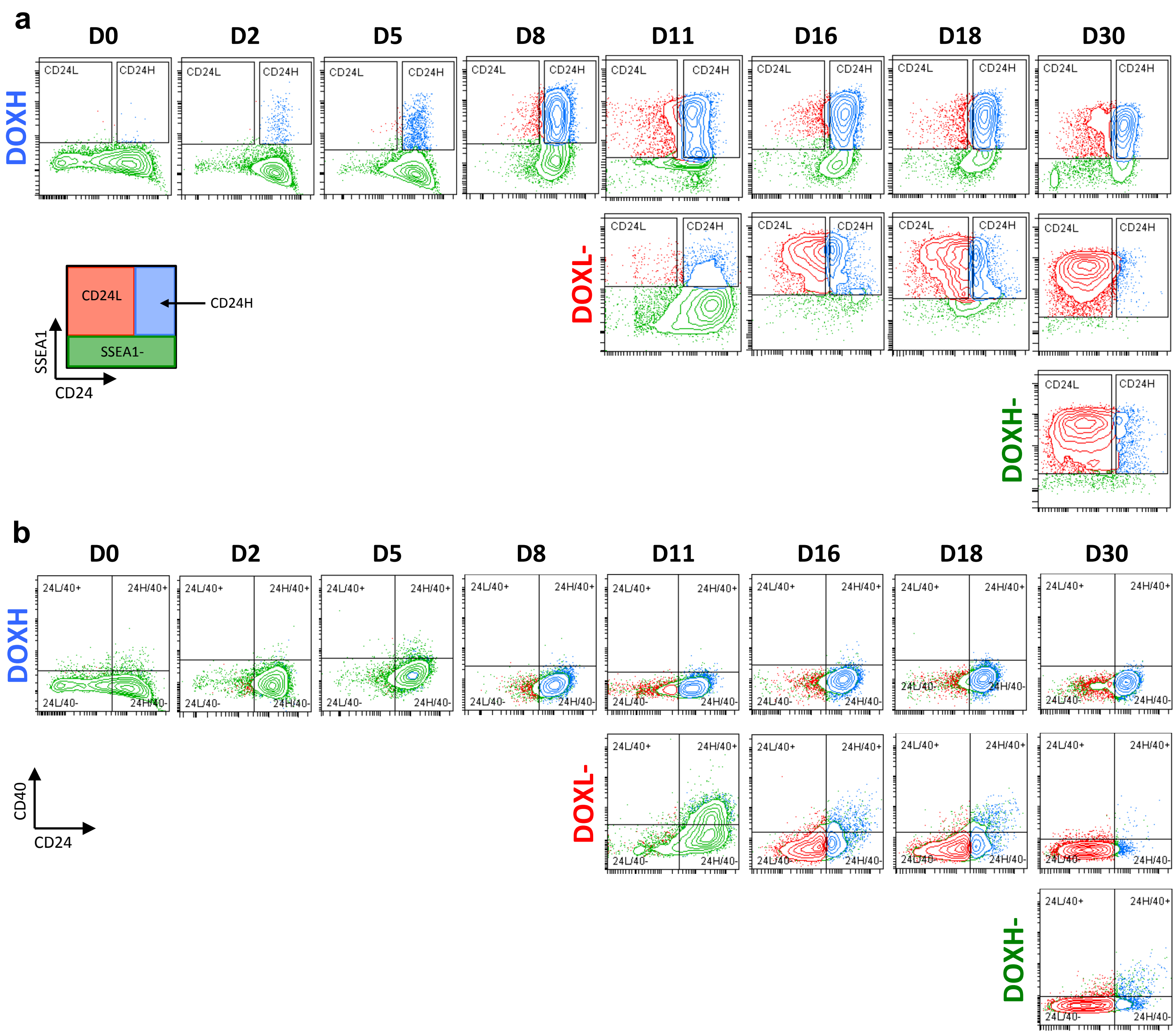

Supplementary Figure 13: CD24/CD40 expression in the 1B tetr reprogramming time course. a, CD24/SSEA1 and **b,** corresponding CD24/CD40 staining of 1B tetr reprogramming time course. Flow plots are representative of 2 biological replicates.

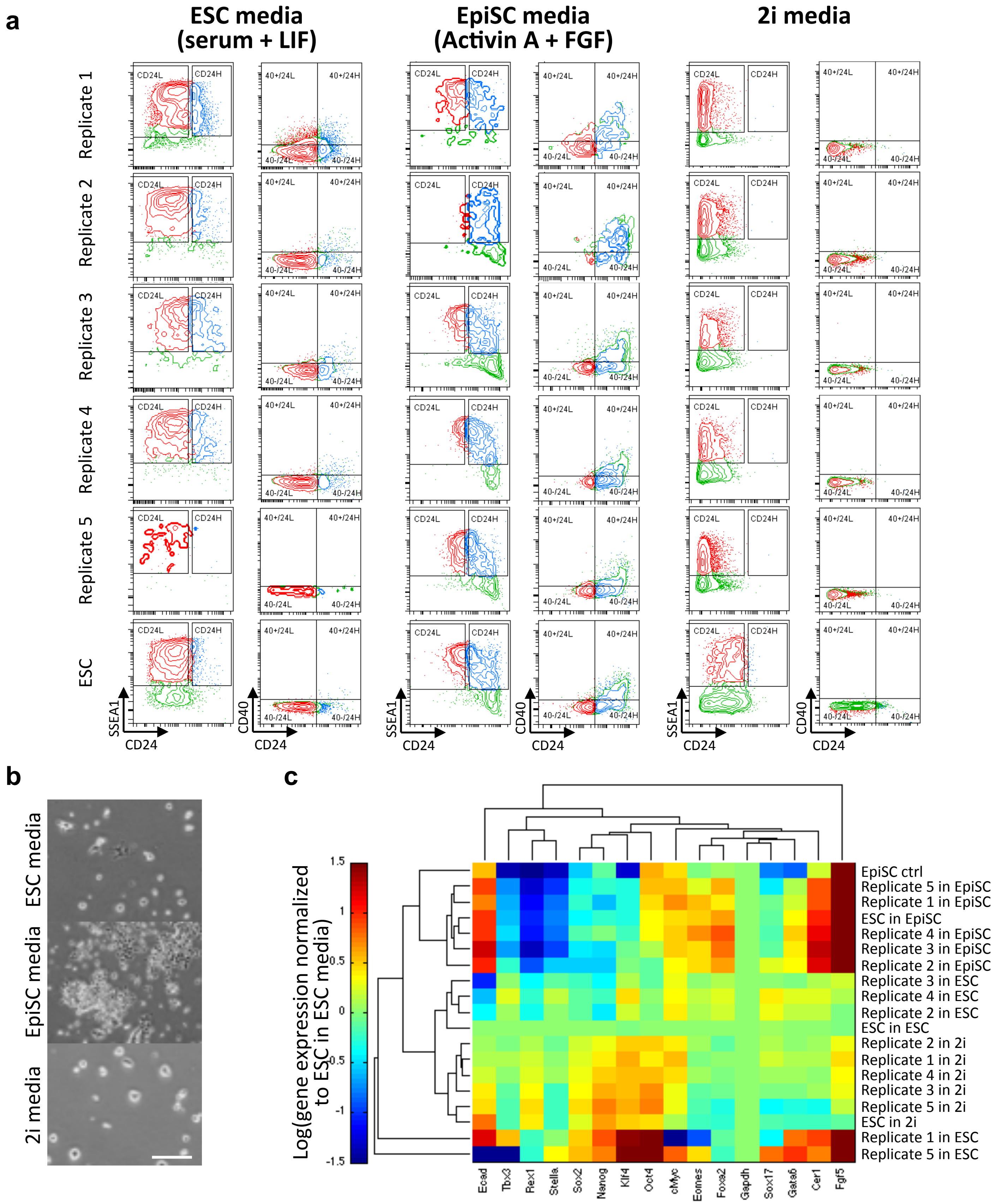

Supplementary Figure 14: Derivation of ESC-like and EpiSC-like cells from ESC-like iPSCs. a, Flow cytometry analysis for CD24/SSEA1/CD40 expression of ESC-like iPSCs derived from 1B reprogramming as well as R1 ESCs cultured in ESC media (serum + LIF), EpiSC media, and 2i media for 18 additional days. Flow plots are representative of 3 technical replicates. **b,** Representative phase contrast images of R1 ESCs following culture in ESC media, EpiSC media, and 2i media for 18 days. Images are representative of 3 technical replicates. Scale bar represents 250 µm. **c**, Hierarchical clustering of gene expression data comparing ESC-, EpiSC-, and 2i-treated iPSCs and R1 ESCs to control embryo-derived EpiSCs. Gene expression normalized to *Gapdh*.

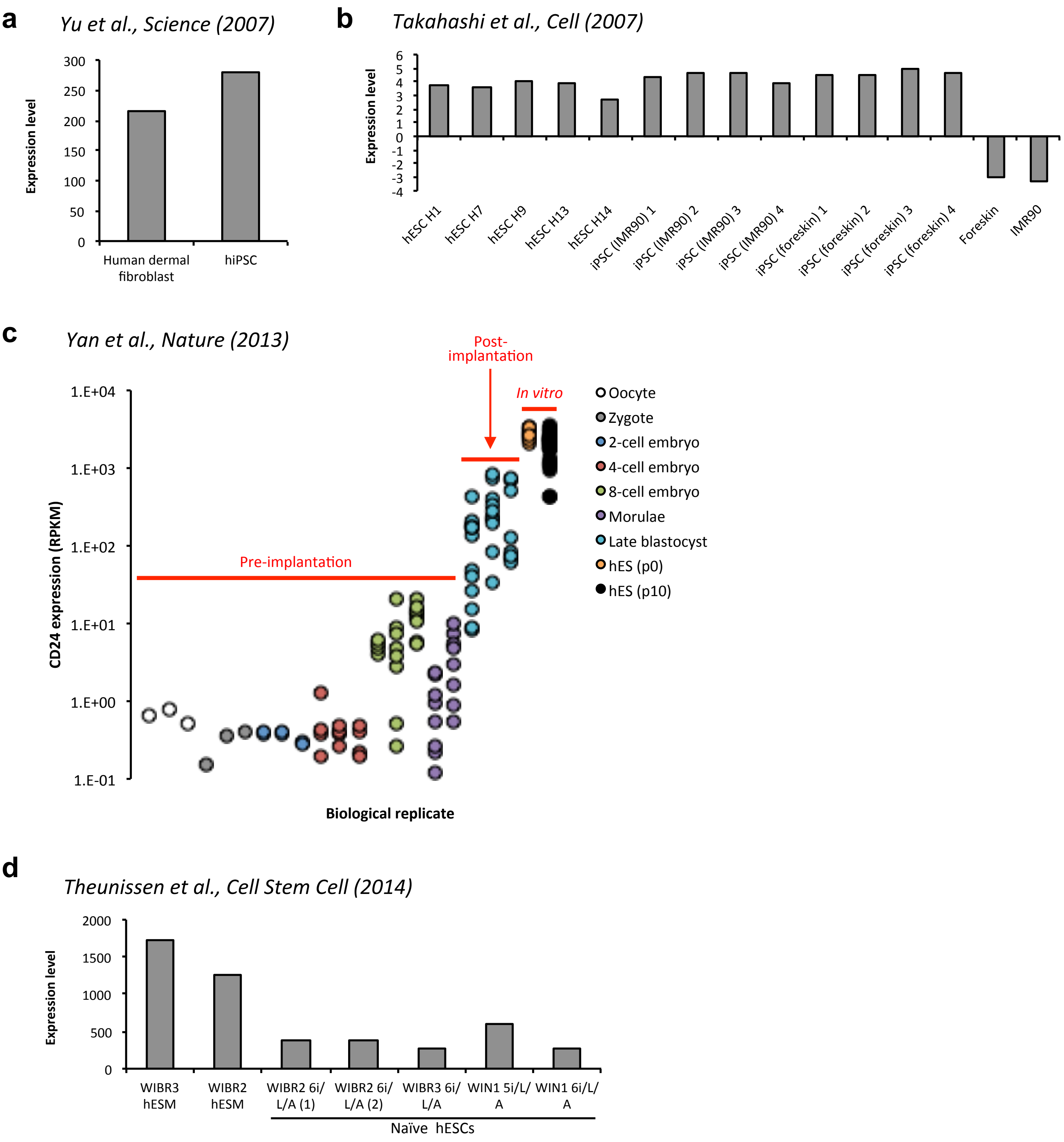

Supplementary Figure 15: CD24 expression in "primed" and "naïve" human pluripotent cells from published reports. a,b, CD24 expression levels extracted from published microarray data characterizing human iPSCs [27,28]. **c,** CD24 expression levels derived from single cell RNA-Seq of the developing human embryo, reported by Yan *et al.*[30]. **d,** CD24 expression levels extracted from published microarray data comparing standard hESCs vs. naïve hESCs[31].

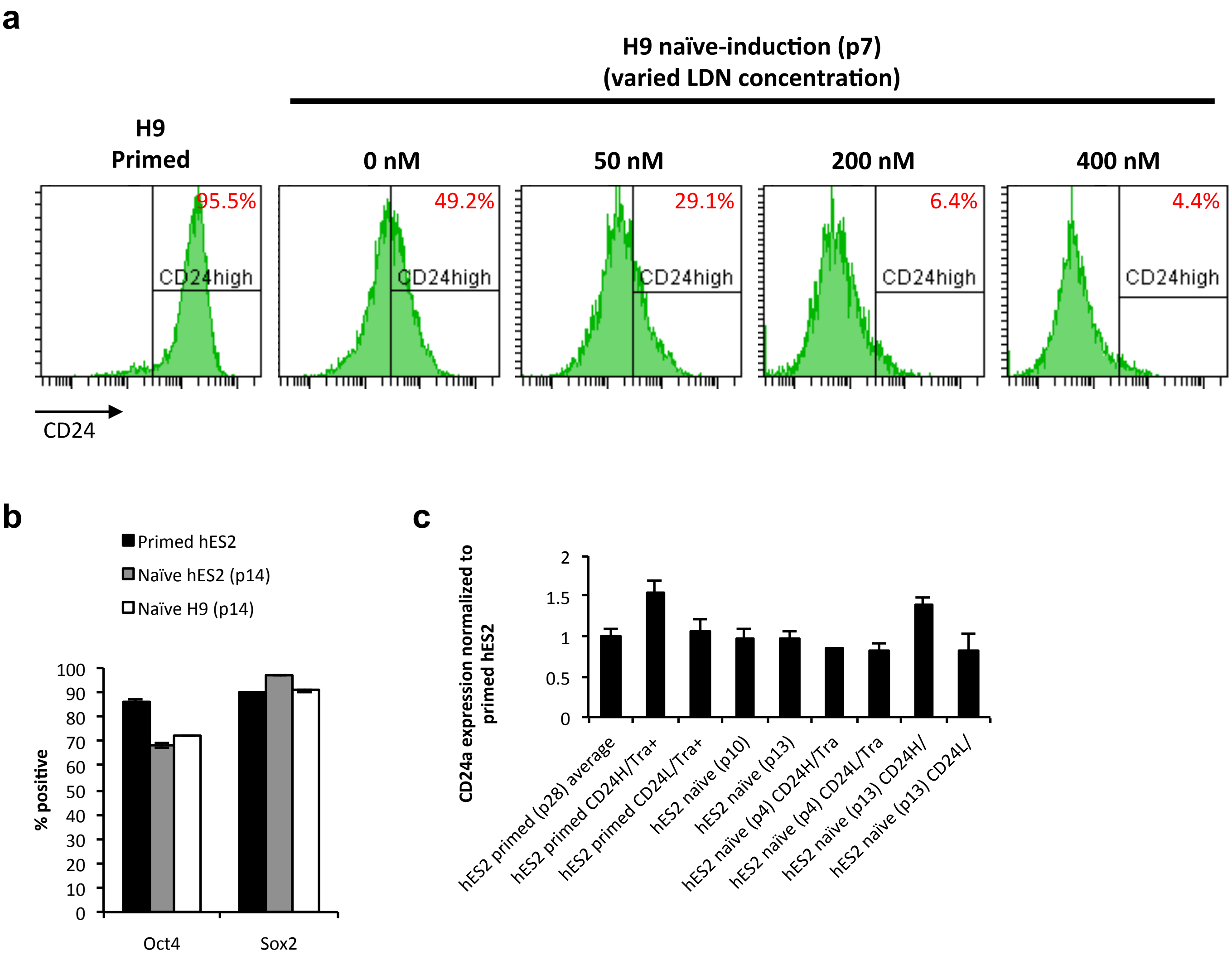

Supplementary Figure 16: Characterization of naïve-induced hESCs. a, Flow cytometry plots showing CD24 expression in H9 hESCs after naïve-induction with varied LDN concentration (p7). Plots are representative of 1 biological replicate. **b,** Flow cytometry analysis for Oct4/Sox2 expression in primed and naïve-induced hES2 and H9 cells (p14). Data bars show mean±s.d. (n=3 technical replicates). **c,** Gene expression level of *CD24a* in naive and primed samples following CD24 sorting. Data bars show mean±s.d. (n=3 technical replicates).

Supplementary Table 1: Sequences of oligonucleotides used in this study.

qPCR Primers

Mouse

Name	Forward	Reverse
Gapdh	AGGTCGGTGTGAACGGATTTG	TGTAGACCATGTAGTTGAGGTCA
Thy1	TTACCCTAGCCAACTTCACCACCA	AAATGAAGTCCAGGGCTTGGAGGA
Alp	CAGTATGAATTGAATCGGAACAACC	CAGCAAGAAGAAGCCTTTGAGG
Oct4	AGTTGGCGTGGAGACTTTGC	CAGGGCTTTCATGTCCTGG
Nanog	TTGCTTACAAGGGTCTGCTACT	ACTGGTAGAAGAATCAGGGCT
Sall4	CCCTGGGAACTGCGATGAAG	TCAGAGAGACTAAAGAACTCGGC
Rex1	CCCTCGACAGACTGACCCTAA	TCGGGGCTAATCTCACTTTCAT
Ecad	CAGGTCTCCTCATGGCTTTGC	CTTCCGAAAAGAAGGCTGTCC
Pecam1	ACGCTGGTGCTCTATGCAAG	TCAGTTGCTGCCCATTCATCA
Dnmt3b	AGCGGGTATGAGGAGTGCAT	GGGAGCATCCTTCGTGTCTG
Dnmt1	ATCCTGTGAAAGAGAACCCTGT	CCGATGCGATAGGGCTCTG
Dppa3/Stella	GACCCAATGAAGGACCCTGAA	GCTTGACACCGGGGTTTAG
Esrrb	AACCGAATGTCGTCCGAAGAC	GTGGCTGAGGGCATCAATG
Oct4_endo	CCATGCATTCAAACTGAGGCACCA	AGCTATCTACTGTGTGTCCCAGTC
Nkx2-3	ACCACCGCAGTGAGATCGAAAG	CGGACAGGTCTTGGATTTGCTCAG
Klf4	AGCCACCCACACTTGTGACTATG	CAGTGGTAAGGTTTCTCGCCTGTG
cMyc	TCCACCGCCGATCAGCTGGA	TGGCAGCGGCTGAGAAACCG
Insml	ATGCCTTGACCTGTTGTCTGTTGG	TGAGGCAGTTACTACAGCATCTCG
Sox2	GCTCGCAGACCTACATGAAC	GCCTCGGACTTGACCACAG
Fetub	AGAGGCACCATGTACCAAATCCC	CTTGGCAAATACCAACGGGCTCAG
Foxa2	CCCTACGCCAACATGAACTCG	GTTCTGCCGGTAGAAAGGGA
Eomes	GGCCCCTATGGCTCAAATTCC	CCTGCCCTGTTTGGTGATG
Gata6	GGCAGTGTGAGTGGAGGTG	CCTGTCTTCTCTTTCGGGTTCA
Sox17	GATGCGGGATACGCCAGTG	CCACCACCTCGCCTTTCAC
Cer1	CTCTGGGGAAGGCAGACCTAT	CCACAAACAGATCCGGCTT
Fgf5	TGTGTCTCAGGGGATTGTAGG	AGCTGTTTTCTTGGAATCTCTCC

Human

Name	Forward	Reverse
Klf4	GCGCTGCTCCCATCTTTCT	GGGGGAAGTCGCTTCATGT
Klf5	CCTGGTCCAGACAAGATGTGA	GAACTGGTCTACGACTGAGGC
Esrrb	ATCAAGTGCGAGTACATGCTC	CGCCTCCGTTTGGTGATCTC
Oct4	TGGGCTCGAGAAGGATGTG	GCATAGTCGCTGCTTGATCG
Nanog	GATTTGTGGGCCTGAAGAA	CAGATCCATGGAGGAAGGAA
Dppa5	ACTCTCCCGGCACGTAGAC	AGGGATTCGAGATCCGTCCG
Dppa2	GGTGCCAGTTAAAGATGACGC	GAGGCAAAATGGTCGGCAAG
Ecad	CCCACCACGTACAAGGGTC	CTGGGGTATTGGGGGCATC
Dnmt3b	AGGGAAGACTCGATCCTCGTC	GTGTGTAGCTTAGCAGACTGG
Stella	CACAAATGCTCACCGAAGAA	AGCTTCCGATAGAGGGGAAA

Sox2	CACTGCCCCTCTCACACATG	TCCCATTTCCCTCGTTTTTCT
GAPDH	GTGGACCTGACCTGCCGTCT	GGAGGAGTGGGTGTCGCTGT
Cd24a	CTCCTACCCACGCAGATTTATTC	AGAGTGAGACCACGAAGAGAC
Rex1	AACGGGCAAAGACAAGACAC	GCTGACAGGTTCTATTTCCGC
LIF-R	TTTCCAGTGGCTGTTATCAACAT	TCCAGAGGGTGCTTTCCAAGA
Dnmt3a	AGTACGACGACGACGGCTA	CACACTCCACGCAAAAGCAC
Otx2	CAAAGTGAGACCTGCCAAAAAGA	TGGACAAGGGATCTGACAGTG
Tbx3	GTGTCTCGGGCCTGGATTC	GCTTCCGAAAGGGGACATGG
Klf2	CACCAAGAGTTCGCATCTGA	GGCTACATGTGCCGTTTCAT

Supplementary Table 2: Antibodies used in this study.

Antibodies

Name	Company	Catalog
Alexa Fluor® 555 anti-Mouse SSEA-1	BD Pharmingen	560119
APC Anti-Mouse CD24	BD Pharmingen	562349
Brilliant Violet 421 Anti-Mouse CD24	BD Pharmingen	562563
Alexa Fluor® 647 anti-human CD24	BD Pharmingen	561644
Anti-Mouse CD54 (ICAM-1) PE	BD Pharmingen	553253
Brilliant Violet 421 Anti-Mouse CD44	BD Horizon	563970
Brilliant Violet 421 Anti-Mouse CD40	BD Horizon	562846
PE Anti-Mouse CD31 (PECAM-1)	eBioscience	12-0311-83
PE E-cadherin	BD Pharmingen	562526
Alexa Fluor® 647 anti-mouse Nanog	BD Pharmingen	560279
Cy3 Smooth muscle alpha-actin	Sigma	C6198
Anti-human Sox17	R&D Systems	AF1924
Mouse anti-beta-III-tubulin	Sigma	T8660
Anti-Mouse SSEA-1	eBioscience	14-8813-82
Anti-Mouse Nanog	eBioscience	14-5761-80
Alexa Fluor® 647 anti-mouse IgG2b	Life Technologies	A21242
Alexa Fluor® 647 goat anti-Mouse IgM (u chain)	Life Technologies	A21238
Alexa Fluor® 647 goat anti-Rat IgG (H+L)	Life Technologies	A21247
Alexa Fluor® 568 anti-Goat IgG	Life Technologies	A11057
Pacific Blue Annexin V	Life Technologies	A35136
